# Putative Prostate Cancer Risk SNP in an Androgen Receptor‐Binding Site of the Melanophilin Gene Illustrates Enrichment of Risk SNPs in Androgen Receptor Target Sites

**DOI:** 10.1002/humu.22909

**Published:** 2015-10-19

**Authors:** Huajie Bu, Narisu Narisu, Bettina Schlick, Johannes Rainer, Thomas Manke, Georg Schäfer, Lorenza Pasqualini, Peter Chines, Michal R. Schweiger, Christian Fuchsberger, Helmut Klocker

**Affiliations:** ^1^ Department of UrologyDivision of Experimental UrologyMedical University of InnsbruckInnsbruckAustria; ^2^ Research Institute for Biomedical Aging ResearchUniversity of InnsbruckInnsbruckAustria; ^3^ Medical Genomics and Metabolic Genetics BranchNational Human Genome Research InstituteNational Institutes of HealthBethesdaMaryland; ^4^ OncotyrolCenter for Personalized Cancer MedicineInnsbruckAustria; ^5^ Biocenter InnsbruckSection for Molecular PathophysiologyMedical University of InnsbruckInnsbruckAustria; ^6^ Center for BiomedicineEURAC ResearchBolzanoItaly; ^7^ Max Planck Institute for Molecular GeneticsBerlinGermany; ^8^ Max Planck Institute for Immunobiology and EpigeneticsFreiburgGermany; ^9^ Department of PathologyMedical University of InnsbruckInnsbruckAustria; ^10^ Cologne Center for GenomicsUniversity of CologneGermany; ^11^ Department of BiostatisticsUniversity of MichiganAnn ArborMichigan

**Keywords:** prostate cancer, risk SNPs, androgen receptor, melanophilin, *MLPH*, androgen regulation, AR

## Abstract

Genome‐wide association studies have identified genomic loci, whose single‐nucleotide polymorphisms (SNPs) predispose to prostate cancer (PCa). However, the mechanisms of most of these variants are largely unknown. We integrated chromatin‐immunoprecipitation‐coupled sequencing and microarray expression profiling in *TMPRSS2‐ERG* gene rearrangement positive DUCaP cells with the GWAS PCa risk SNPs catalog to identify disease susceptibility SNPs localized within functional androgen receptor‐binding sites (ARBSs). Among the 48 GWAS index risk SNPs and 3,917 linked SNPs, 80 were found located in ARBSs. Of these, rs11891426:T>G in an intron of the melanophilin gene (*MLPH*) was within a novel putative auxiliary AR‐binding motif, which is enriched in the neighborhood of canonical androgen‐responsive elements. T→G exchange attenuated the transcriptional activity of the ARBS in an AR reporter gene assay. The expression of MLPH in primary prostate tumors was significantly lower in those with the G compared with the T allele and correlated significantly with AR protein. Higher melanophilin level in prostate tissue of patients with a favorable PCa risk profile points out a tumor‐suppressive effect. These results unravel a hidden link between AR and a functional putative PCa risk SNP, whose allele alteration affects androgen regulation of its host gene *MLPH*.

## Introduction

Prostate cancer (PCa) is the most prevalent cancer and one of the leading causes of cancer‐related death in Western men [Siegel et al., 2012]. In 56% of primary tumors and in almost all tumor metastases, androgen receptor (AR) is overexpressed and deregulated [Taylor et al., 2010]. Therefore, AR signaling has been both a main target for therapy and a focus in research for understanding the molecular mechanisms of PCa development and progression.

AR regulates a wide variety of genes involved in cell proliferation, migration, invasion, and apoptosis. Recently, the identification of AR targets in both PCa cell lines and tumor tissues has been greatly extended by high‐throughput techniques, such as gene expression profiling and chromatin‐immunoprecipitation (ChIP) coupled with microarray analysis or DNA sequencing (ChIP‐seq) [Lin et al., 2009; Massie et al., 2011; Sharma et al., 2013]. These approaches enable the identification of key signal pathways, as well as clarification of the changes of AR signaling during PCa tumorigenesis and progression. A discovery of great importance disclosed by genetic analysis of prostate tumors was the identification of gene rearrangements involving the promoter regions of androgen‐regulated genes. Most frequent among these genetic alterations in PCa are fusions of *ETS* transcription factor genes to 5′‐regions of AR‐regulated genes, which results in androgen‐stimulated overexpression of ETS proteins [Tomlins et al., 2005].

Among a variety of fusion genes in PCa, the *TMPRSS2‐ERG* fusion is the most common one with a prevalence of 40%–70% in primary tumors [Schaefer et al., 2013]. ERG overexpression was reported to increase stemness of prostate tumor cells [Casey et al., 2012]; however, the full functional importance and clinical implications of the fusion gene remain to be unraveled. Interestingly, ERG was also found to inhibit transactivation of the AR via direct and indirect mechanisms, thus modulating AR signaling in ERG fusion gene‐positive cancers [Yu et al., 2010]. Despite extensive studies on the AR transcriptome and cistrome, these have been largely focused on LNCaP cells, which do not harbor a *TMPRSS2‐ERG* fusion gene. There is a need, therefore, to study the deregulated AR signaling in fusion gene‐positive PCa tumor cells.

Genome‐wide association studies (GWAS) have been widely applied to identify the association of common genetic variants with cancer risk. Single‐nucleotide polymorphisms (SNPs) in several genetic loci such as 8q24, 22q13 and 17q12 were reported to be linked to PCa susceptibility, early onset of the disease, and tumor aggressiveness [Witte, 2007; Levin et al., 2008; Salinas et al., 2008; Thomas et al., 2008; Chang et al., 2009; Cheng et al., 2009; Eeles et al., 2009; Gudmundsson et al., 2009; Takata et al., 2010; Schumacher et al., 2011; Eeles et al., 2013; Al Olama et al., 2014; Berndt et al., 2015; Helfand et al., 2015]. Although little is known about the functional aspect of risk SNPs, some studies showed cancer SNPs predominately present in multiple putative regulatory elements [Sille, et al., 2012]. SNPs in the promoter of the *KLK3* gene, encoding the commonly used PCa marker protein prostate‐specific antigen (PSA), were reported to increase serum PSA and *PSA* promoter activity [Cramer et al., 2003], whereas a C→ T substitution of SNP rs10993994:C>T in the 5′ region of the PCa‐suppressor gene *MSMB* was shown to affect gene expression level [Chang et al., 2009]. By combining GWAS susceptibility genes with expression profiling studies, genes involved in cytoskeleton and cell adhesion were found to be overrepresented among the PCa risk genes [Gorlov et al., 2009]. This finding indicates the feasibility to identify causal variants that regulate the candidate genes and the molecular mechanisms of tumor risk modulation by integrating high‐throughput datasets, for example, GWAS and gene expression profiling.

In this study, by coupling AR ChIP‐seq and microarray expression profiling of androgen‐regulated genes, we identified multiple AR regulatory elements (AREs) in the *TMPRSS2‐ERG* fusion gene‐positive DUCaP PCa cells and identified a novel auxiliary AR‐binding motif enriched in the vicinity of canonical androgen response elements. Correlation with GWAS data revealed enrichment of PCa risk SNPs in AR‐binding sites (ARBSs). A common SNP, rs11891426:T>G, which is in moderately high‐linkage disequilibrium with the GWAS SNPs rs2292884:A>G and rs7584330:A>G, was found located within one of the auxiliary ARE motifs within the ARBS in the seventh intron of the *MLPH* gene (OMIN accession number: *606526). We further showed that the variant allele of rs11891426:T>G is negatively correlated with melanophilin (MLPH) expression. A higher protein expression in prostate tissues of cancer patients was associated with a favorable PCa risk profile, suggesting a causal relationship between PCa development and progression with modulation of *MLPH* expression.

## Materials and Methods

### Cell Culture

Human PCa cell lines LNCaP and PC‐3 were obtained from ATCC (Manassas, VA). DUCaP was a generous gift from Dr. Jack Schalken (Center for Molecular Life Science, The Netherlands). LNCaP cells were originally derived from a lymph node metastasis of a PCa patient (Horoszewicz et al., 1983). Their AR harbors a point mutation in the ligand‐binding domain, leading to a promiscuous receptor activated by estrogens, progestins, and by flutamide in addition to androgens [Kokontis et al., 1991]. DUCaP PCa cells were obtained from a dura mater metastasis of a PCa patient [Lee et al., 2001] and harbor a *TMPRSS2‐ERG* gene rearrangement (found in 50%–70% of all prostate tumors). These cells express a high level of wild‐type AR. LNCaP and DUCaP cells are both androgen sensitive, whereas PC‐3 cells, originally isolated from a bone metastasis of a PCa patient [Kaighn et al., 1979], do not express AR and are androgen unresponsive [Sampson et al., 2013]. Introduction of AR into PC‐3 cells through transient transfection of an expression vector, however, restores AR signaling and response to androgens. LNCaP cells were cultured in RPMI 1640 (PAA, Pasching, Austria) supplemented with 10% fetal bovine serum (FBS; PAA), 2 mM l‐glutamine (Invitrogen, Carlsbad, CA), 2.5 g/l d‐glucose (Invitrogen), 10 mM HEPES, and 1 mM Na‐Pyruvat (Lonza, Basel, Switzerland). PC‐3 and DUCaP cells were maintained in RPMI 1640 with 10% FBS and 2 mM l‐glutamine. Before steroid hormone treatment cells were held in phenol‐red‐free RPMI 1640 medium (Fisher, Logan, UT) supplemented with 10% charcoal/dextran‐treated fetal calf serum (CCS; Fisher) for up to 3 days. Stimulation with 1 nM of the synthetic androgen R1881 or vehicle was performed for the indicated durations.

### Clinical Samples

Patients’ tissue samples for DNA, RNA, and protein analysis were obtained from the Prostate Cancer Bioresource of the Department of Urology, Innsbruck Medical University. The clinical samples have been collected within the framework of the Tyrolean prostate cancer early detection program [Bartsch et al., 2008]. Written informed consent was obtained from all patients and the study was approved by the Ethics Committee of the Innsbruck Medical University (Study AM 3174 and amendment 2). All samples were obtained from patients with biopsy‐proven, clinically localized PCa, who were at least 40 years old and who had received no previous PCa therapy. A total of 126 patients were enrolled in the study. For 108 cases, DNA genotyping was performed, mRNA expression was determined in 77 cases, and 68 cases were available for immunohistochemistry analysis. Patients’ variables such as age, total PSA, percent‐free PSA, prostate tumor volume, and clinical and histological tumor characteristics are recorded in Table 1.

**Table 1:** Clinicopathological Variables of Enrolled Prostate Cancer Patients

Variable	Number of patients	Mean	Range
Age at diagnosis (years)	125	62	41–76
Total PSA (ng/ml)	123	5.42	1.35–54.7
Free PSA (% of total PSA)	100	13.05	1.42–39.13
Tumor Volume (ml)	42	1.08	0.24–12.9
			Number of Patient
Gleason score		≤ 3+4	75
		≥ 4+3	54
Pathological stage		T2a+T2c	76
		T3a+T3b+T4	50
Estimated tumor mass (%)		<10%	56
		≥10%	34
Biochemical recurrence (PSA rise)		Yes	32
		No	50

### ChIP‐Coupled Deep Sequencing

ChIP‐seq was performed as described [Bu et al., 2013]. AR antibodies used for ChIP were from Cell Signaling (Danvers, MA) and Upstate (PG‐21, UB 06‐680). Rabbit IgG was used as antibody control. After adjustment to steroid hormone‐free condition, DUCaP cells were treated with 1 nM R1881 or vehicle control and cells were harvested at different time points and subjected to ChIP. The enrichment of AR to its target genes at different time points was monitored by normal or qPCR amplification of well‐established AR target sites, for example, the AR enhancer in the PSA gene promoter (F: 5′‐GGGGTTTGTGCCACTGGTGAG‐3′ and R: 5′‐GGGAGGCAATTCTCCATGGTTC‐3′). AR enrichment was highest after 1 hr of hormone treatment and this time point was chosen for CHIP‐seq analysis. Samples including appropriate control samples (vehicle control, IgG control ± androgen) were prepared once and DNA was immunoprecipitated and purified. After a quality check using qPCR as described above, DNA samples were used for library preparation and sequenced on a Solexa platform (Illumina Genome Analyzer II; Illumina, San Diego, CA).

### Identification of CHIP‐Seq Peak Regions and AR Target Genes

Raw sequencing data were analyzed by the Illumina analysis pipeline software and reads were aligned to the unmasked human reference genome (NCBI v36, hg18) using Bowtie [Langmead et al., 2009]. All reads that mapped to simple tandem repeats [Benson, 1999] or duplicated regions >1,000 bp were discarded. MACS tool (v1.4) was used to identify AR‐enriched regions in a genome‐wide manner in comparison to control IgG‐enriched regions [Zhang et al., 2008]. AR‐enriched regions in androgen‐treated samples were compared with control vehicle‐treated samples to identify androgen‐dependent AR‐binding peaks. The AR‐binding peaks with −10log10 *P* values more than 50 were counted as ARBSs. If an ARBS occurred within 50 kb upstream or downstream of a gene start site or within the body of a gene, we regarded that gene as a potential AR target gene. ChIP‐seq data have been deposited to the Gene Expression Omnibus (accession Nr. GSE70679).

### Expression Profiling

DUCaP or LNCaP cells were steroid starved for 3 days before treatment with 1 nM R1881 or equivalent ethanol for 8 or 24 hr. Total RNA was isolated using TRI reagent (Sigma, St. Louis, MO) according to the manufacturer's instructions. Biological triplicate (24‐hr treatment) and duplicate (8‐h treatment) RNAs were hybridized to Affymetrix HuGene‐1.0 st v1 expression arrays (Affymetrix, Santa Clara, CA) [Irizarry et al., 2003] in the Core Facility of the Medical University of Innsbruck. Raw microarray data were preprocessed in R (http://www.r‐project.org) using the RMA algorithm and the custom CDF HuGene10stvs1_Hs_ENSG version 12 [Smyth, 2004]. Raw and preprocessed microarray data have been deposited to the Gene Expression Omnibus (accession Nr. GSE63693). Significance for differential expression was estimated using the moderated *t*‐test and the resulting *P* values were adjusted for multiple hypotheses testing using the Benjamini and Hochberg correction method [Benjamini and Hochberg, 1995]. Probe sets with a B‐H‐corrected *P* value ≤ 0.05 were considered to represent androgen‐regulated genes.

### Characterization of Androgen‐Responsive Elements and AR‐Binding Motifs

MEME (version 4.70) was run with default options, allowing detected motif sites to be on forward or reverse strand (‐revcomp). A file of 100 bp sequences centered around the binding peaks of 5,571 ARBSs of the 1,490 AR primary targets was used as the input to the program. Novelty of the detected motifs was determined based on comparison of the motifs against those in JASPAR using TomTom [Gupta et al., 2007]. This study utilized the high‐performance computational capabilities of the Biowulf Linux cluster at the National Institutes of Health, Bethesda, MD (http://biowulf.nih.gov).

### Linkage of GWAS Data and ARBSs

SNPs reported to be associated with PCa (*P* value <5×10^−8^) were obtained from the NHGRI GWAS catalog (http://www.genome.gov/gwastudies, accessed Dec 31, 2011) and defined as GWAS index SNPs. Linked SNPs were identified as those that have ≥specified *r* ^2^. For simulation analysis, *r* ^2^ = 0.2, 0.5, and 0.7 were used, whereas *r* ^2^ ≥ 0.5 was chosen for further analysis of SNPs linked with the index SNPs based on the genotypes of the 1000G project (phase 1, v3). For analysis of GWAS SNPs enrichment in the AR ChIP‐seq peaks, 10,000 random sets of simulated ChIP‐seq peaks, matching chromosome, and size of the actual peaks were picked across the genome. The number of the index and linked SNPs in the random regions was counted and compared against the observed one in ChIP‐seq peaks. A permutation *P* value was estimated using Wilson score interval [Wilson, 1927] to quantify the significance of enrichment of the index and linked SNPs overlapping the ARBSs compared with the ones identified in simulated peaks.

### Reporter Vector Construction and Site‐Directed Mutagenesis

Firefly luciferase reporter vectors harboring the ARBS of the *MLPH* gene, with the major T allele of SNP rs11891426:T>G, were constructed according to the procedures previously described [Bu et al., 2013]. Briefly, the *MLPH* ARBS was PCR amplified using primers F: 5′‐ TATCCAACACACGGGCTGAT ‐3′ and R: 5′‐ AGCTTTGGGGATTTCATTTCA ‐3′, purified and first ligated to the PCR fragment cloning vector PSC‐B (Agilent Technologies, Santa Clara, CA), and then inserted into the KpnI/SacI sites of the PGL3 promoter luciferase reporter plasmid (Promega, Madison, WI). The minor G allele counterpart of the reporter vector or mutations of the two putative androgen‐responsive elements (AREs) were generated using the QuikChange II site‐directed (Agilent) or the Q5 site‐directed mutagenesis (NEB, Ipswich, MA) kits. Primers used for mutagenesis were 5′‐CAGCTCCCTGCT**G**CCAGCCTGGGG for G allele alteration, 5‐GCTTCCAGCCTGGGGTG**C**G**TTC**TGCA**C**GCCTCCCTGAAATG for mutation of AR binding motif 3 and 5′‐GCCAGCCCACAGC**GTT**CTGC**ACG**CAGCCTGGGGTGGGAC for mutation of AR‐binding motif 2.

### Luciferase Reporter Gene Assay

AR‐negative PC‐3 cells were seeded at a density of 50% confluence in a 96‐well plate. On the next day, they were transfected with 40 ng of AR expression vector PSG5‐AR together with 40 ng of the firefly reporter vectors harboring either the wild‐type ARBS or a mutated counterpart and 4 ng of renilla luciferase normalization control vector (PGL4.73; Promega). AR‐positive DUCaP cells were seeded at a density of 175,000 cells per well in 24‐well plates and transfected with 0.5 μg per well of one of the firefly reporter vectors and a SV40 promoter‐driven renilla luciferase normalization control vector. One day following transfection, cells were treated with 1 nM R1881 for 48 hr, followed by cell lysis and quantification of luciferase activity using the Dual Luciferase Reporter Assay (Promega). Firefly luciferase activity was normalized to renilla luciferase control activity.

### RNA Isolation from Benign and Malignant Prostate Tissue and qPCR

Total RNA was extracted from carefully macrodissected benign and malignant areas of frozen tissue sections derived from radical prostatectomy specimens (*n* = 77), using the AllPrep DNA/RNA Micro Kit (Qiagen GmbH, Hilden, Germany). RNA concentrations and purity were determined spectrophotometrically. Reverse transcription was performed on 500 ng of total RNA using the iScript select cDNA synthesis kit (Bio‐Rad, Hercules CA) and random hexamer primers (Promega). qPCR control samples for monitoring assay insensitivity for DNA contaminations were prepared by omitting reverse transcriptase during cDNA synthesis.

Quantitative PCR (40 cycles) was performed in triplicates using 8 ng total RNA equivalents of cDNA for each 10 μl reaction. Samples without cDNA and reverse transcription control samples were included to control for unspecific signals. The following primer/probe combination or Taqman assays (Applied Biosystems, Foster City, CA) were used: AR: 5′‐AGGATGCTCTACTTCGCCCC‐3′ (fwd), 5′‐ACTGGCTGTACATCCGGGAC‐3′ (rev), 5′‐TGGTTTTCAATGAGTACCGCATGCACA‐3′ (probe), MLPH: Hs00225445_m1; TBP: Hs00427620_m1. Target gene expression was normalized against the reference housekeeping gene TBP. Standard curves for each PCR assay were generated with a serially diluted calibrator sample (cDNA mix of three benign and three malignant tissue sections, 100, 20, 2, 0.2, 0.02 ng RNA equivalents). The relative expression ratio (*R*) was computed based on assay efficiency of target gene and reference gene and the Ct deviation of each sample to the calibrator sample using the following formula: *R* = (*E*_target_^⋀^Δ*C*_target_[calibrator – sample])/(*E*_reference_^⋀^ΔCt_reference_[calibrator – sample]) (Pfaffl, 2001).

### DNA Isolation and SNP Analysis

Genomic DNA was isolated from carefully macrodissected benign and malignant areas of frozen tissue sections (*n* = 108) using the AllPrep DNA/RNA Micro Kit (Qiagen GmbH). DNA concentrations and purity were determined spectrophotometrically. SNP analysis was performed in triplicate on 3 ng/μl DNA using the 7900HT‐Real Time PCR System. SNP genotyping assays (GenXpress, Vösendorf, Austria) designed against reference sequences rs11891426:T>G, rs2292884:A>G, rs7584330:A>G were applied. Calling rate was 97.87%.

### Tissue Microarray and Immunohistochemistry

Protein expression in malignant and nonmalignant (benign) PCa tissue was analyzed using a tissue microarray (TMA). For the TMA construction, formalin‐fixed and paraffin‐embedded (FFPE) human tissue samples derived from radical prostatectomy (*n* = 68) were selected. From each prostate specimen, three cancer and three benign tissue cores were punched and assembled in a TMA using a manual tissue arrayer (Beecher Instruments, Sun Prairie, WI). A double immuostaining was performed on 5 μm sections using antibodies directed against cytokeratin 5/6 and α‐methylacyl‐CoA racemase (AMACR) or MLPH and AR on a Discovery‐XT staining device (Ventana, Tucson, AZ). Target antibodies and concentrations used were as follows: anti‐cytokeratin 5/6 1:300, anti‐AMACR 1:300 (#M7237, #M3616; Dako, Glostrup, Denmark), anti‐MLPH 1:200 (HPA014685; Sigma), and anti‐AR 1:400 (#3165‐1; Abcam, Cambridge, UK). Images were acquired using an Axio Imager Z2 microscope (Zeiss, Jena, Germany) and TissueFAXS software (TissueGnostics, Vienna, Austria) and MLPH and AR immunostaining intensity analysis was performed using the HistoQuest IHC analysis software (TissueGnostics) under the supervision of an experienced uropathologist (GS). Cytokeratin 5/6/AMACR stains were used to control assignment of benign and malignant tissue areas. For each TMA spot, the mean intensity and percentage of positively stained cells were evaluated and a score was calculated by multiplying those two values. The mean immunointensity score value for each patient was calculated as the average of the three tissue cores, stratified according to cancer and benign tissue.

### Statistical Analysis

The GraphPad Prism 5 (Graph Prism Software Inc., La Jolla, CA) software package was used for statistical analysis. Numerical data are presented as the mean and standard deviation of at least three independent experiments. The differences of the relative luciferase activities between the reporter vectors harboring wild‐type and mutated sequences were calculated using the unpaired *t*‐test. Differences of *MLPH* expression levels according to different tissue types, genotypes, as well as patients’ variables and tumor characteristics (age, total PSA, % free PSA, histopathological grade and stage, tumor volume, tumor percentage, and PSA recurrence) were analyzed using the Welch‐corrected unpaired *t*‐test. The Pearson's test was used to calculate the correlation of AR and MLPH protein or mRNA expression and the Chi‐square test to estimate differences of the distribution of *MLPH* genotypes among subgroups of patients. *P* values of ≤0.05 were considered statistically significant and are indicated in graphs as *P ≤ 0.05, **P ≤ 0.01, and ***P ≤ 0.001.

## Results

### Genomic AR Targets in DUCaP PCa Cells

In this study, we used DUCaP cells as a model to investigate the AR‐regulated transcriptional program in PCa cells. This cell line expresses a high level of wild‐type AR, which mimics AR upregulation seen in the majority of androgen‐refractory PCa [Edwards et al., 2003]. Furthermore, it harbors a *TMPRSS2‐ERG* gene rearrangement found in 40%–70% of prostate tumors [Schaefer et al., 2013]. Overexpression of the encoded ERG fusion protein was reported to modulate AR signaling [Yu et al., 2010].

The dynamics of androgen‐dependent AR enrichment to its target genes was monitored at different time points (0, 20 min, 1, 4, and 18 hr) following R1881 treatment by PCR or qPCR amplification of a PSA enhancer fragment from isolated ChIP‐DNA. As expected, androgen treatment induced a recruitment of AR to the target site. Highest enrichment was seen at the early time points (20 min and 1 hr) and it decreased afterwards (4 and 18 hr; Supp. Fig. S1). One hour androgen treatment was therefore chosen as the time point for the identification of genome‐wide ARBS by ChIP‐deep sequencing analysis.

AR ChIP‐seq of androgen‐stimulated DUCaP cells identified 39,156 ARBSs. The MACS *P* values of the AR binding peaks are shown in Supp. Figure S2A. In vehicle‐treated DuCaP cells only one ARBS in the *CDK12* gene proximal promoter was identified. Further validation using independent ChIP samples could not confirm this site, indicating a false‐positive ChIP‐seq signal (data not shown). These results indicated the dependence on androgen stimulation for receptor activation and genomic targeting by the AR. The DUCaP genomic ARBSs were compared with Lin's ChIP‐seq dataset of PC3‐AR cells [Lin et al., 2009]. Of the 4,165 ARBSs identified in PC3‐AR cells, 2,804 (67%) overlapped with our dataset and a further comparison with Massie's ChIP‐seq data of LNCaP cells [Massie et al., 2011] revealed 8,899 of 11,054 (80.5%) overlapping ARBSs. Of the 51,811 total VCaP cell line binding sites published in the same study, 29,823 (58%) were in common with the DUCaP ARBSs. These results support the presence of common AR genomic‐targeted sites even in distinct molecular subtypes of PCa. To identify those ARBSs that are actively involved in transcription, we first integrated the DUCaP genomic AR sites with DNaseI hypersensitivity chromatin sites across a range of cell types (table wgEncodeRegDnaseClustered V1 of http://genome.ucsc.edu; UW ENCODE). Fifty‐nine percent (23,155) of the 39,156 ARBSs overlapped with this marker for transcriptionally active chromatin.

**Figure 1:**
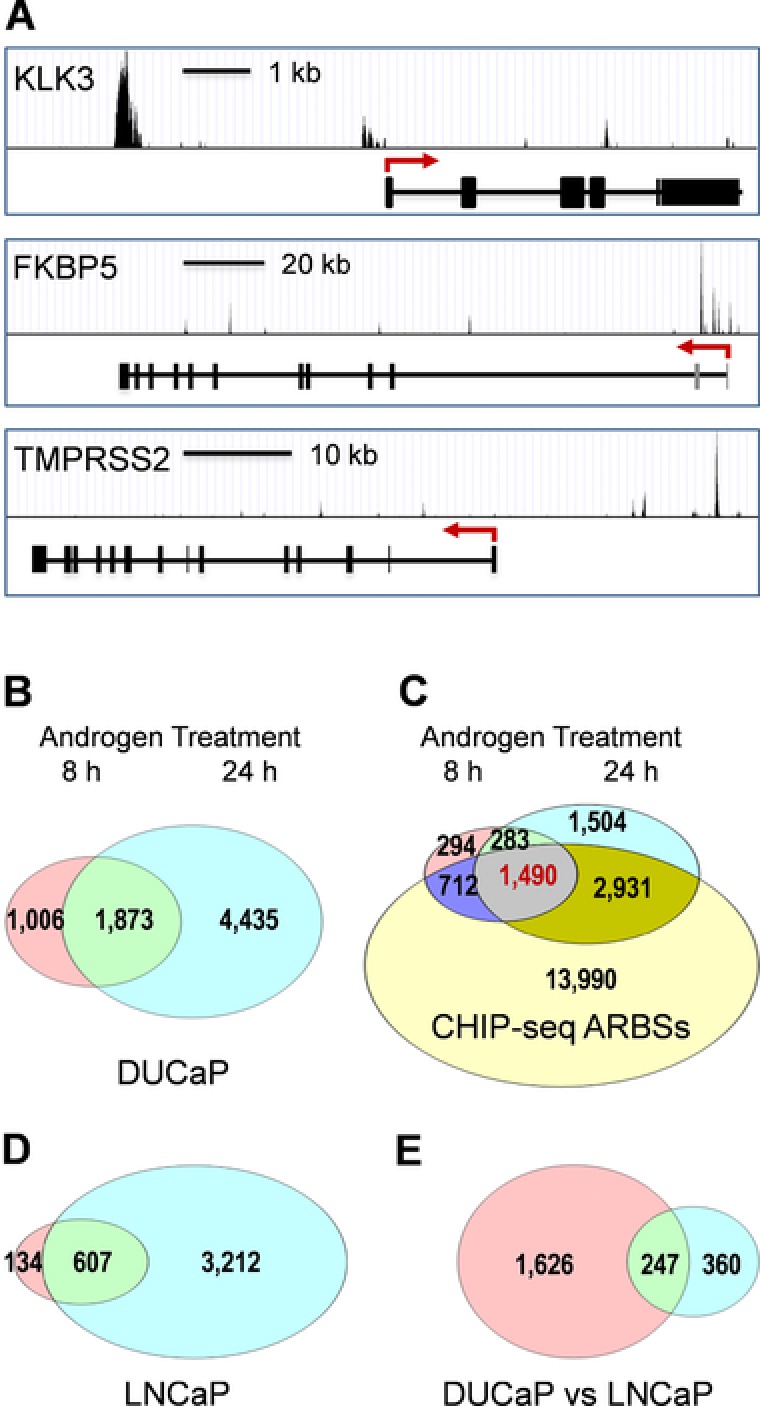
Identification of primary AR target genes in DUCaP cells. ChIP‐seq and gene expression microarray data obtained from androgen‐stimulated DUCaP cells formed the basis to identify genomic AR target sites (ARBSs) and the androgen‐regulated transcriptome of DUCaP cells. **A**: Validation of ChIP‐seq data. Schematic illustration of AR target sites showing ARBSs in the regulatory regions of prototype androgen‐responsive genes, *KLK3* (PSA), *FKBP5*, and *TMPRSS2*, identified by ChIP‐seq. **B**: Venn diagram illustrations of androgen‐regulated genes following 8 and 24 hr of hormone (1 nM R1881) treatment in DUCaP cells. **C**: Venn diagram identifying genes that harbor ARBSs and are androgen regulated in the same direction in DUCaP cells stimulated for 8 or 24 hr. These 1,490 genes were designated as the primary AR target genes. **D**: Genes regulated by androgen stimulation at both time points in LNCaP cells are shown as overlaps. **E**: Comparison of androgen‐regulated genes in DUCaP with those in LNCaP cells.

We next defined potential AR target genes based on the distance to ARBSs. Regulatory sites targeted by AR can reside quite a distance upstream from the transcription start sites of target genes or lie within the gene regions [Jariwala et al., 2007; Massie et al., 2011]. Therefore, we considered a gene to be an AR target if it was located within a 50‐kb distance to an ARBS or an ARBS was located within the gene. A total of 19,123 UCSC annotated genes fulfilled these criteria. Notably, well‐known androgen‐regulated genes such as KLK3, FKBP5, and TMPRSS2 [Cleutjens et al., 1997; Wang et al., 2007; Makkonen et al., 2009) were called by our analysis algorithm (Fig. 1A).

### Identification of Primary AR Target Genes in DUCaP Cells

The androgen‐induced transcriptional program was determined in DUCaP cells using Affymetrix gene array expression profiling. Androgen‐regulated genes were identified following 8 and 24 hr of androgen stimulation. The early time point was chosen in an attempt to identify the primary AR response genes. Using a BH‐corrected *P* value cutoff of ≤0.05, a total of 2,879 and 6,308 out of the 17,837 genes probed on the microarray were regulated upon androgen stimulation of 8 and 24 hr, respectively. Two thirds of genes regulated after 8 hr (65%, 1,871 genes) were also modulated in the same direction after 24 hr of treatment (Fig. 1B). The majority of genes regulated at either time point harbor ARBSs (8 hr: 76%, 24 hr: 70%) indicating that AR binding is a prerequisite for androgen‐mediated regulation in most of these genes (Fig. 1C). Considering both time points, 1,873 genes were regulated in the same direction, either up or down (Fig. 1B; Supp. Table S1). When these genes were integrated with the genomic AR target site genes, 80% of them (1,490 genes) overlapped indicating that these are primary AR target genes (Fig. 1C; Supp. Table S1).

One‐third of the 1,490 primary AR target genes (478) overlapped with androgen‐regulated genes harboring ARBSs in PC3‐AR cells (Wang et al., 2009). The comparison with Massie's list of androgen‐regulated genes in LNCaP cells showed an overlap of only 91 genes (6%). We cross‐validated androgen stimulation of gene expression in LNCaP cells using Affymetrix gene array analysis under the same conditions as for DuCaP cells. Of 740 genes regulated after 8 hr, 607 (82%) were also modulated in the same direction after 24 hr (Fig. 1D). Less than half of these (247, 44%) overlapped with DUCaP primary genes (Fig. 1E), which is significantly less than the percentage of shared ARBSs, which showed an overlap of 80.5%. These divergences among different AR‐positive cell lines indicate that AR binding is not the only key player determining androgen‐dependent gene expression. Rather, different cell lines seem to display distinct AR primary target genes despite a high conformity of common ARBSs.

To answer the question whether the distance of ARBSs relative to the transcriptional start sites (TSS) of genes affects androgen regulation, we compared the distribution of ARBS–TSS distances in the DuCaP 1,490 androgen‐regulated primary AR target genes with the distances in all the other genes containing ARBSs. The pattern of distribution is similar in both sets: the ARBSs are preferentially enriched in close proximity to the TSSs of the genes and distances are independent of androgen regulation (Supp. Fig. S2B). In addition, the ARBSs are also relatively evenly distributed at distal enhancer regions within 25 kb upstream or downstream of the TSS. This result is in agreement with several other reports that ARBSs are not limited to the proximal promoter immediately upstream of the TSS [Jariwala et al., 2007; Takayama et al., 2007; Wang, et al. 2007; Waltering et al., 2009].

### Identification of Androgen‐Binding Motifs and Response Elements (AREs)

**Figure 2:**
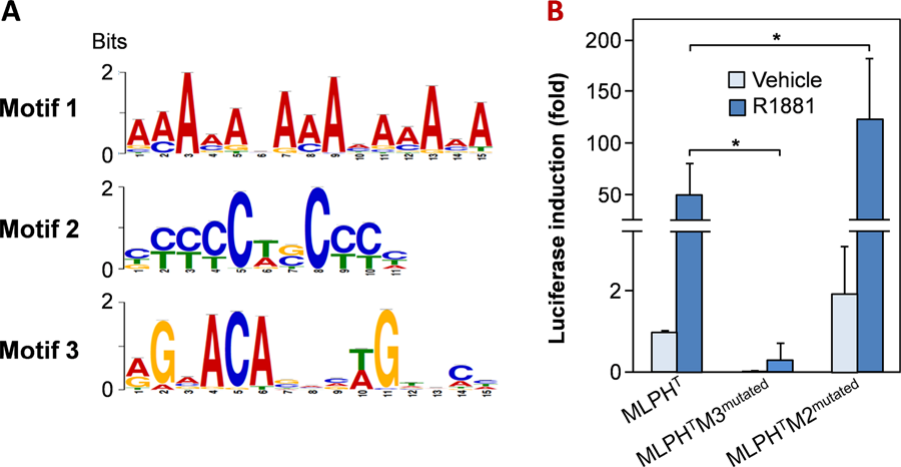
Identification and functional analysis of AR‐binding motifs. **A**: Consensus motifs were extracted from the 5,571 ARBSs of the primary AR target genes and analyzed for frequent motifs. The top three motifs are shown. Of these, the 15‐bp motif 3 has a high homology to a consensus androgen‐responsive element (ARE), whereas motif 2 represents a novel 11 bp CG‐rich element and motif 1 a 15‐bp AT‐rich AR‐binding element. **B**: The ARBS in the 7^th^ intron of the *MLPH* gene harbors a motif 2 element followed by a motif 3 ARE element at a distance of 11 bp (see Supp. Fig. S4.). This ARBS with wild‐type major T allele of rs11891426:T>G in motif 2 was cloned into the enhancer site of the PGL3P firefly luciferase reporter vector as described in experimental procedures and the vector was designated “MLPH^T^.” Its counterparts with mutations abolishing either motif 2 or 3 were constructed using site‐directed mutagenesis and referred to as “MLPH^T^M2^mutated^” and “MLPH^T^M3^mutated^.” The firefly luciferase reporter vectors together with the renilla luciferase transfection control vector PGL4.73 were transfected into DUCaP cells and stimulated by androgen. The androgen‐induced changes in luciferase activity were measured after treatment with 1 nM R1881 or vehicle for 48 hr using a dual luciferase assay. The firefly luciferase activity was normalized to the transfection control renilla luciferase activity and compared relative to the activity of the unstimulated MLPH^T^ vector. Reporter gene assay values represent mean+SD of at least three independent experiments. Statistical differences were calculated using the unpaired *t*‐test. *P < 0.05.

We further characterized the 5,571 ARBSs of the 1,490 AR primary targets and searched for common sequence motifs and putative androgen‐responsive elements. Using MEME [Bailey et al., 2009], we took 100 bp sequences centered at the ARBS peaks to identify potential ARE motifs. Three motifs are consistently found in high frequency across different parameter settings with an incidence of 622, 1,085, and 971 for motif 1, 2, and 3, respectively (Fig. 2A). Among them, TomTom [Gupta et al., 2007] comparison showed that only motif 3 has a high level of similarity to a consensus ARE, namely, Ar (id MA0007.1; [Roche et al., 1992]) in the JASPAR database (http://jaspar.binf.ku.dk) (Fig. 2A), which is described in the Transfac database as well (id V$AR_01) (http://www.gene‐regulation.com/pub/databases.html). The other two motifs are not associated with any known AREs in both databases. Fourteen percent of motif 1 (*n* = 87) and 13.7% of motif 2 (*n* = 149) are within 100 bp distance to a motif 3. Considering the random coincidence rates that estimated 1.9% coenrichment for motif 1 and 3, and 3.3% for motif 2 and 3, respectively, this indicates a nonrandom clustering of the motifs. An analysis of the distance of the motifs to the TSS of the nearest genes shows that motif 2 is preferentially enriched around TSSs, followed by motif 3, whereas motif 1 seems to be randomly distributed in the genetic context (Supp. Fig. S2C).

### Association of AR Genomic Targets with PCa Risk

The recent advances in GWAS have greatly extended our knowledge of genetic loci related to human disease risk and genetic traits. SNPs associated with a disease or a phenotype are enriched within noncoding functional elements such as transcription factor‐binding sites [Ortiz‐Barahona et al., 2010; ENCODE Project Consortium et al., 2012]. We asked the question whether PCa risk loci colocalize with ARBSs. For that purpose, GWAS PCa risk index SNPs and their linked SNPs were retrieved from the NHGRI GWAS catalog ([Hindorff et al., 2009], http://www.genome.gov/gwastudies) and linked to ARBSs. Of the 48 GWAS index PCa risk SNPs and their 3,917 linked SNPs (*r* ^2^≥0.5 defined by the 1000G project phase 1, v3), 80 were found to be localized in ARBSs (Supp. Table S2). We recognized a SNP to be overlapping with an ARBS if it was located anywhere in the binding peak region called by the peak calling software MACS. Simulation of random ChIP‐seq peaks showed that the observed ChIP‐seq peaks are highly enriched with GWAS risk SNPs and their linked SNPs, as none of 10,000 simulated sets of random peaks across the genome achieved more than the observed number of risk SNP overlaps (permutation *P* value 0.00024, Wilson score 95%, confidence interval 0–0.00038; Supp. Fig. S3). Different *r* ^2^ cutoffs (0.7 and 0.2) in the analysis showed the same level of enrichment of GWAS SNPs in ARBSs. This indicates a nonrandom enrichment of GWAS PCa risk index and linked SNPs in ARBSs.

**Figure 3:**
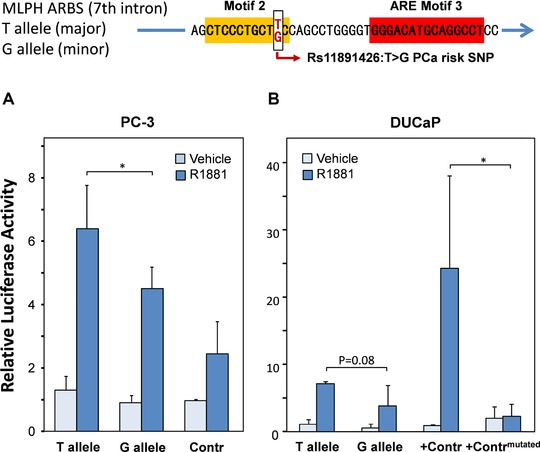
Impact of the putative PCa risk SNP rs11891426:T>G on androgen‐regulated gene expression. The *MLPH* ARBS cloned into the luciferase reporter vector contained the major T allele of the NHGRI GWAS PCa putative risk SNP rs11891426:T>G in the ARE motif 2. The G allele counterpart of the MLPH ARBS reporter was constructed by site‐directed mutagenesis. **A‐B**: The reporter vectors were transfected together with the AR expression vector pSG5AR into AR‐negative PC‐3 cells (**A**), or without the AR expression vector into DUCaP cells (**B**). The renilla luciferase vector PGL4.73 was used to normalize for transfection efficiency and the empty PGL3P vector served as a baseline control (Contr) in PC‐3 cells. As a positive control (+Contr), a known androgen‐regulated reporter vector containing the AGR3 gene enhancer, which harbors a consensus ARE motif 3 [Bu et al., 2013] and its mutated counterpart (+Contr^mutated^) were employed in DUCaP cells. The androgen‐induced changes in luciferase activity were measured after 48 hr of treatment with 1 nM R1881 using a dual luciferase assays. The firefly luciferase activity was normalized to the transfection control renilla luciferase activity and compared relative to the activity of the unstimulated baseline control in PC‐3 cells (**A**), or to the positive control vector in DUCaP cells (**B**). Reporter gene assay values represent mean+SD of at least three independent experiments. Statistical differences were calculated using the unpaired *t*‐test. *P < 0.05.

Integration of the GWAS risk and linked SNPs with the ARBSs of the 1,490 primary AR target genes revealed 20 SNPs localized in ARBSs of five androgen‐regulated genes (Table 2). Among them, the SNP rs11891426:T>G (G is the risk allele) is located within the putative AR‐binding motif 2 of the respective ARBS in the 7^th^ intron of the *MLPH* gene (OMIN accession number: *606526). Exchanging T→G abolishes the motif 2. In addition, this MLPH–ARBS contains a putative motif 3 ARE, just in 11 bp distance from the motif 2 element (Fig. 3). rs11891426:T>G has a *r* ^2^ of 0.37 with the reported PCa risk SNP rs7584330:A>G (*G* is risk allele) (Kote‐Jarai, et al., 2011a) and a *r* ^2^ of 0.53 with rs2292884:A>G (*G* is risk allele), another PCa risk SNP [Haiman et al., 2011] (Supp. Fig. S4). Although *MLPH* is in close proximity to the PCa‐associated SNPs, a possible molecular mechanism for disease causality has not yet been reported. These findings prompted us to further investigate the influence of the two ARE motifs and their resident SNP rs11891426:T>G on MLPH gene regulation.

**Table 2:** SNPs Localized in ARBSs of Primary Androgen‐Regulated Genes in DuCaP Cells

Number	SNP	Chr.	Position	GWAS index SNP	*r* ^2^ with GWAS index SNP	Gene	Reference
1	rs11883500	chr2	238,099,173	rs2292884	0.61	MLPH	Schumacher, et al. (2011)
2	rs11890255	chr2	238,099,344	rs2292884	0.61	MLPH	Schumacher, et al. (2011)
3	rs11890307	chr2	238,099,444	rs2292884	0.61	MLPH	Schumacher, et al. (2011)
4	rs11891426	chr2	238,099,441	rs2292884	0.53	MLPH	Schumacher, et al. (2011)
5	rs7558685	chr2	238,099,682	rs2292884	0.51	MLPH	Schumacher, et al. (2011)
6	rs7598035	chr2	238,099,681	rs2292884	0.51	MLPH	Schumacher, et al. (2011)
7	rs60676526	chr17	44,809,379	rs7210100	1.00	ZNF652	Haiman, et al. (2011)
8	rs12474519	chr2	238,041,751	rs7584330	0.84	MLPH	Kote‐Jarai, et al. (2011)
9	rs13386290	chr2	238,057,875	rs7584330	0.88	MLPH	Kote‐Jarai, et al. (2011)
10	rs6737914	chr2	238,058,173	rs7584330	0.96	MLPH	Kote‐Jarai, et al. (2011)
11	rs6740722	chr2	238,058,027	rs7584330	0.50	MLPH	Kote‐Jarai, et al. (2011)
12	rs80151891	chr2	238,063,565	rs7584330	0.50	MLPH	Kote‐Jarai, et al. (2011)
13	rs112162741	chr12	51,560,766	rs902774	1.00	KRT8, EIF4B, TENC1	Schumacher, et al. (2011)
14	rs4919737	chr12	51,557,062	rs902774	0.84	KRT8, EIF4B, TENC2	Schumacher, et al. (2011)
15	rs4919740	chr12	51,557,238	rs902774	0.83	KRT8, EIF4B, TENC3	Schumacher, et al. (2011)
16	rs4919743	chr12	51,595,851	rs902774	0.82	KRT8, EIF4B, TENC4	Schumacher, et al. (2011)
17	rs73108429	chr12	51,557,938	rs902774	0.84	KRT8, EIF4B, TENC5	Schumacher, et al. (2011)
18	rs902772	chr12	51,560,040	rs902774	1.00	KRT8, EIF4B, TENC6	Schumacher, et al. (2011)
19	rs902773	chr12	51,560,046	rs902774	1.00	KRT8, EIF4B, TENC7	Schumacher, et al. (2011)
20	rs902774	chr12	51,560,171	rs902774	1.00	KRT8, EIF4B, TENC8	Schumacher, et al. (2011)

### The effect of AR‐Binding Motifs and Putative PCa Risk SNP rs11891426:T>G on Gene Expression

The MLPH gene expression was enhanced upon androgen treatment for 24 hr by 2.2‐fold in LNCaP cells and 1.8‐fold in DuCaP cells according to our gene array expression data. In order to analyze the functional importance of the novel AR‐binding motif 2 and the canonical motif 3 within the ARBS on AR transcriptional activity, luciferase reporter vectors harboring motifs 2 and 3, and its flanking sequences (illustrated in Supp. Fig. S5) were constructed and mutations that abolish motifs 2 or 3 were introduced as well. In AR‐positive DUCaP cells, androgen treatment significantly induced the reporter gene containing the MLPH major allele ARBS element (MLPHwt‐T) over nonstimulated cells (49‐fold induction; Fig. 2B). Mutation of motif 3 in the ARBS completely abolished the androgen regulation of the reporter gene, whereas destruction of motif 2 did not, and even increased androgen regulation (125‐fold induction; Fig. 2B). This result indicated the prerequisite of ARE motif 3 for androgen regulation, whereas motif 2 seems to modulate androgen regulation by motif 3.

To investigate the effect of the minor G allele in the putative risk SNP rs11891426:T>G on androgen regulation, the reporter vector containing G at position 10 of ARE motif 2 was constructed (Supp. Fig. S4). In PC‐3 cells, in which androgen regulation was re‐established by AR overexpression, androgen treatment induced the wild‐type T reporter gene significantly by 6.4‐fold, whereas the risk allele G reporter gene was induced by 4.5‐fold only (Fig. 3A). In DUCaP cells, similar differences, although less significant, were seen (Fig. 3B). This result indicated that the presence of the risk G allele in the ARE motif 2 attenuates AR‐dependent regulation of MLPH gene expression.

### Correlation of the rs11891426:T>G Genotype with *MLPH* Expression in PCa Tissues

The link of the risk G allele of rs11891426:T>G with reduced AR transactivation activity in vitro prompted us to further study the association of the SNP genotype with MLPH expression in PCa tissues. We genotyped rs11891426:T>G, as well as two associated SNPs, rs7584330:A>G and SNP rs2292884:A>G, in prostate tissue DNA samples of 108 PCa patients. For the majority of cases, paired benign and tumor DNA samples were available and the genotypes were identical in all paired samples. The detected minor allele frequencies were 13% for rs11891426:T>G, 21% for rs2292884:A>G, and 21% for rs7584330:A>G.

**Figure 4:**
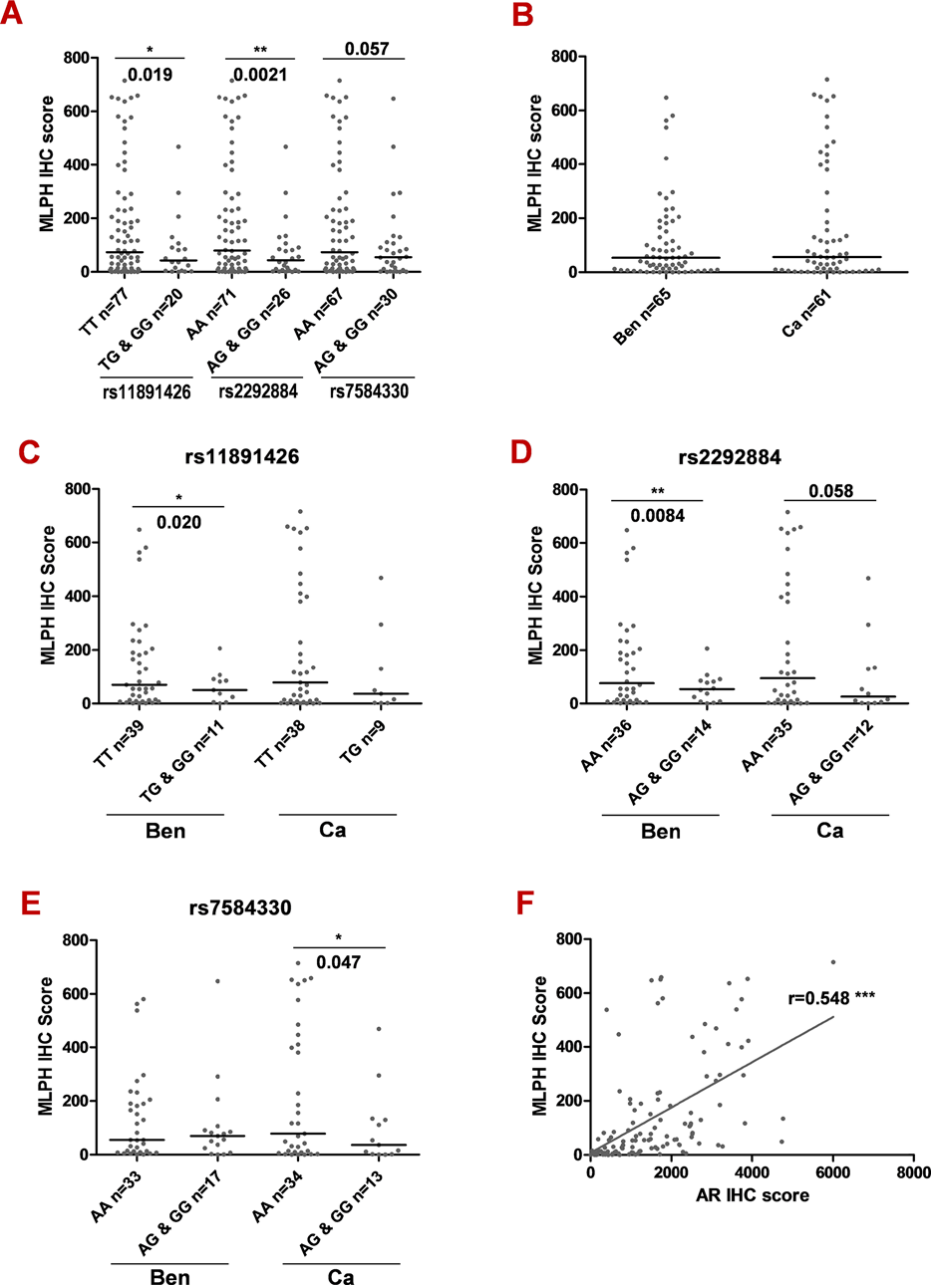
Correlation of MLPH protein expression to SNP genotypes and to AR expression in PCa tissue. MLPH and AR protein expression was analysed by immunohistochemistry using a TMA, which contained benign (*n* = 65 cases) and cancer (*n* = 61 cases) FFPE tissue samples obtained from radical prostatectomy specimens (*n* = 68). For each case, three cancer tissue cores and three benign cores were stained using specific antibodies, and staining intensity was analyzed using the HistoQuest immunohistochemistry analysis software. Genomic DNA isolated from benign and malignant areas of frozen tissue sections from 112 patients was genotyped for three SNPs, rs11891426:T>G, rs2292884:A>G, rs7584330:A>G, using genotyping PCR assays. Altogether, for 52 cases, comprising 97 benign or tumor samples, immunoreactivity scores, and genotypes were available for analysis. **A**–**E**: Scatter plots of the MLPH protein expression stratified according to the genotypes of rs11891426:T>G, rs2292884:A>G, and rs7584330:A>G (**A**), benign (Ben) and cancer (Ca) tissue types (**B**), tissue types and genotype categories of rs11891426:T>G (**C**), rs2292884:A>G (**D**), and rs7584330:A>G (**E**). **F**: Scatter plot of AR versus MLPH immunoreactivity scores demonstrating the correlated expression of AR and MLPH protein in prostate tissues. Welch's corrected unpaired *t*‐test was used for the analysis of differences between the groups. *P < 0.05, **P < 0.01, and ***P < 0.001.

In order to correlate the expression of *MLPH* with the distinct SNP allele, MLPH immunohistochemistry analysis was carried out employing a TMA containing benign and tumor tissue cores of radical prostatectomy specimens obtained from 68 patients. Of 52 of them, the genotype was also available. Overall, 126 benign or cancer tissue samples originating from these 68 patients, each sample represented by three tissue cores, were analyzed by IHC and mean immunoreactivity scores were calculated for each case and used for statistical analysis. MLPH protein immunoreactivity was significantly lower in samples harboring a rs11891426:T>G risk G allele (G/G *n* = 1, T/G *n* = 19) compared with those harboring the nonrisk T allele (T/T *n* = 77; *P* = 0.019; Fig. 4A). This confirmed the finding of the in vitro reporter gene assay that the risk G allele may attenuate gene expression (Fig. 3A). Furthermore, the tissue samples with one or two risk G alleles (A/G *n* = 23 or G/G *n* = 3) in SNP site rs2292884:A>G, a GWAS index SNP that has an *r* ^2^ of 0.53 with rs11891426:T>G, also showed a significantly lower expression of the protein when compared with tissue samples with two major A alleles (*n* = 71, *P* = 0.0021; Fig. 4A). The difference was less significant with regard to different alleles of the second GWAS index SNP rs7584330:A>G, which has a correlation coefficient *r* ^2^ of 0.37 with rs11891426:T>G (G/G *n* = 6 and A/G *n* = 24 vs. G/G *n* = 67, *P* = 0.057; Fig. 4A).

Overall, there was no significant difference in the expression of MLPH protein in benign (Ben; *n* = 65) compared with cancer (Ca; *n* = 61) tissues (Fig. 4B). However, in both tissue types, MLPH immunoreactivity was lower in cases with the rs11891426:T>G G (risk) allele when compared with the T allele, with a statistically significant difference for the benign tissue type (*P* = 0.020; Fig. 4C). A similar expression pattern of MLPH was also found for the rs2292884:A>G SNP (*P* = 0.0084 in benign, *P* = 0.058 in tumor tissue; Fig. 4D). For rs7584330:A>G, a lower expression was only evident in tumor tissue (*P* = 0.047; Fig. 4E). To further confirm the influence of AR on *MLPH* expression, we correlated the expression of AR and MLPH proteins in the prostate tissues and found a significant positive correlation of immunoreactivities (*r* = 0.548, *P* < 0.0001; Fig. 4F).

In addition, we investigated the effect of the SNP genotypes on MLPH mRNA expression in prostate tissues by qRT‐PCR. Overall, different alleles of rs11891426:T>G, rs2292884:A>G, and rs7584330:A>G were not associated with significantly different mRNA expression levels (Supp. Fig. S6A). A stratification into benign and cancer‐derived RNA samples showed decreased mRNA expression associated with the minor allels of rs11891426:T>G, rs2292884:A>G, and rs7584330:A>G in cancer tissues; however, a close to statistical significance was obtained only with rs2292884:A>G (Supp. Fig. S6B–D). Again, there was a significant positive correlation of MLPH and AR mRNA levels (*r* = 0.265, *P* = 0.001; Supp. Fig. S6E). Different to protein levels, MLPH mRNA levels in tumor (*n* = 77) were significantly higher compared with benign tissues (*n* = 74) (Supp. Fig. S6F).

### Association of MLPH Genotypes and Protein Expression with Clinicopathological Variables

An analysis of the correlation of *MLPH* genotypes and disease characteristics revealed an association of the AG and GG genotypes of rs7584330:A>G with lower pathological stages (pT2 vs. pT3 and pT4; *P* = 0.021). All other genotypes showed no significant association with any clinicopathological parameters (Supp. Table S3).

**Figure 5:**
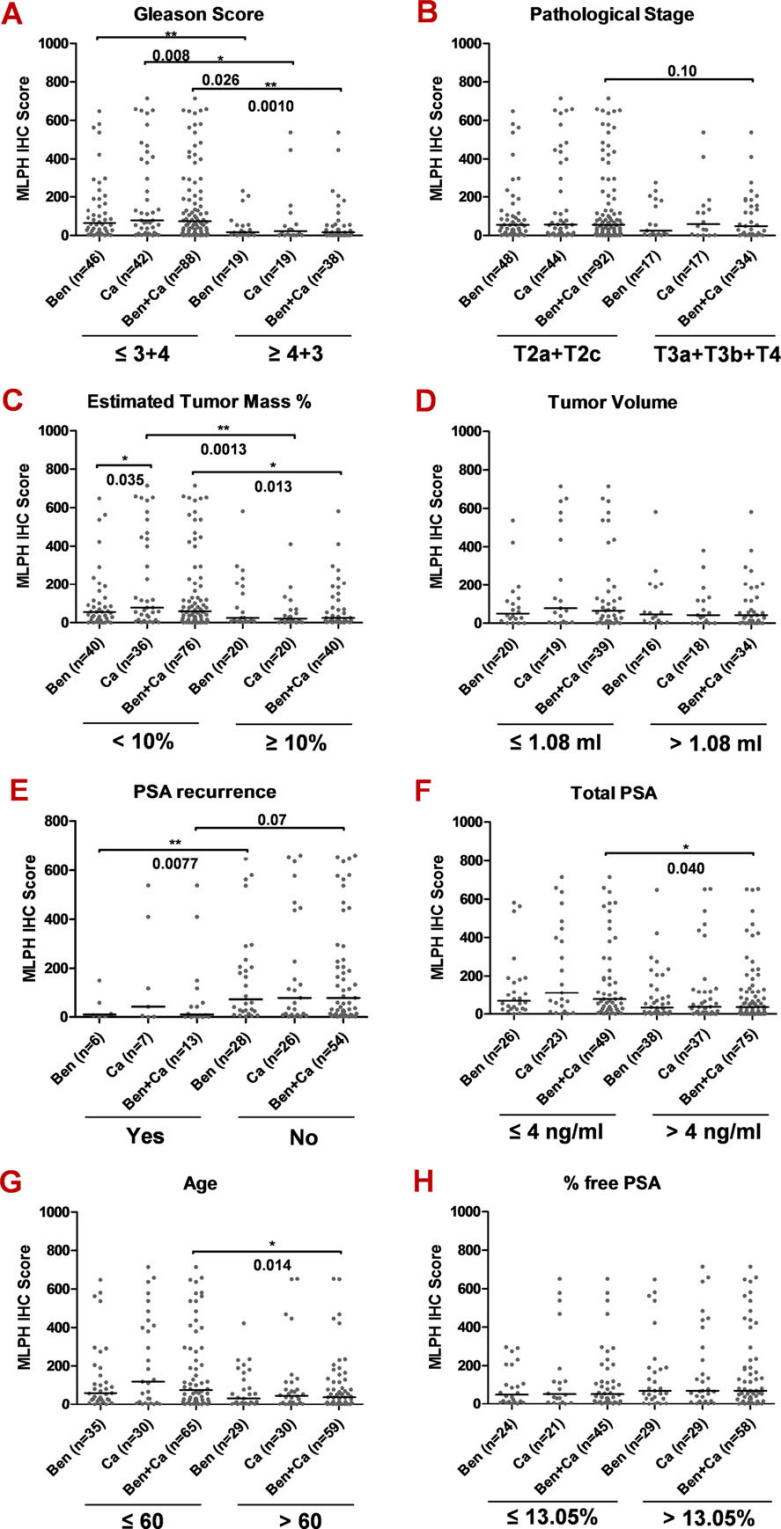
Correlation of MLPH protein expression to clinicopathological parameters. MLPH protein expression in prostate tissue, determined by immunohistochemistry analysis, was stratified according to: tumor Gleason scores (**A**), histopathological stage (**B**), estimated tumor mass (**C**), tumor volume (**D**), PSA (biochemical) tumor recurrence (**E**), serum PSA levels (**F**), patients’ age at tumor diagnosis (**G**), and percentage‐free PSA (**H**). Welch's corrected unpaired t‐test was used for the analysis of differences between the stratification groups. * P < 0.05, ** P < 0.01.

In order to further investigate a possible role of MLPH in PCa, we correlated *MLPH* expression with clinical characteristics and clinical outcome. Higher MLPH immunoreactivity in radical prostatectomy specimens was associated with lower Gleason scores (Gleason score **≤**3+4 vs. ≥4+3; Fig. 5A) and with lower pathological stages (pT2 vs. pT3 and pT4; Fig. 5B), whereby the latter failed to reach statistical significance (*P* = 0.1). A significant higher protein expression was also associated with lower relative tumor mass (tumor mass <10% vs. ≥10%), although the expression level was not associated with tumor volume itself (Fig. 5C and D). In line with these observations, higher immunoreactivity scores were found in patients without a biochemical (PSA) tumor recurrence (statistically significant in benign samples, close to significance in all samples; Fig. 5E), and in patients with a lower serum PSA (≤4 vs. > 4 ng/ml; Fig. 5F). Higher expression was also associated with younger age at tumor diagnosis (≤60 vs. >60 years; Fig. 5G), whereas no association was observed with % free PSA (>13.05% vs. ≤13.05%; Fig. 5H). With regard to MLPH mRNA expression differences, no parameter used for patient stratification showed a significant association (Supp. Fig. 7A–H). Solely within several stratification groups, the expression level was significantly higher in tumor compared with benign tissue.

Taken together, the pattern of MLPH expression reveals higher protein levels mainly associated with lower risk tumor parameters thus indicating a tumor‐suppressive function of this protein in PCa.

## Discussion

Using ChIP‐seq, we profiled genome‐wide androgen‐dependent ARBSs in DUCaP cells, which harbor a TMPRSS2‐ERG gene rearrangement, a genetic alteration found in 40%–70% of prostate tumors [Rubin et al., 2011; Schaefer et al., 2013]. A total number of 39,156 ARBSs were detected in the DUCaP cells, which is markedly higher than the number of binding sites found in the LNCaP cell line and comparable to the binding sites identified in the VCaP cells, a bone‐metastasis counterpart of DUCaP derived from the same patient [van Bokhoven et al., 2003; Massie et al., 2011]. Higher expression levels of AR in DUCaP and VCaP cells were proposed to be responsible for a higher number of genomic binding sites. This is in line with increasing numbers of ARBSs in LNCaP cells manipulated to express higher levels of AR [Urbanucci et al., 2012].

Despite different numbers of ARBSs and different molecular characteristics of LNCaP and DUCaP cells, more than 80% of the LNCaP ARBSs overlapped with DUCaP‐binding sites, indicating that AR binding to chromatin target sites is mainly dependent on genomic sequences and less on the molecular cell characteristics. Interestingly, of the 19,123 genes with ARBSs, only 1,490 were actually identified as primary AR target genes in DUCaP cells upon their uniform regulation after 8 and 24 hr of androgen stimulation. Although there is a high degree of overlap of genes harboring ARBSs in different AR‐positive PCa cell lines, the concordance of actually androgen‐regulated genes among different cellular models is much smaller. For example, less than half of the LNCaP genes we found androgen‐regulated in our gene array analysis were also androgen regulated in the DUCaP cells, suggesting a more pronounced influence of molecular properties and cellular phenotype on the actually androgen‐regulated gene set.

The distribution of ARBSs in DUCaP cells is similar in both androgen‐responsive and androgen‐unresponsive genes, indicating that other mechanisms, for instance, location of ARBS‐harboring genes in regions of either active or inactive chromatin, play a crucial role in addition. Several interacting transcription factors that interrupt AR signaling might also account for this unexpected finding. The TMPRSS2‐ERG fusion gene present in DUCaP cells was reported to disrupt AR signaling [Yu et al., 2010], and forkhead protein FoxA1 can modulate AR function through either pioneering or masking the pathway [Sahu et al., 2011].

A comparative analysis of all ARBSs in the authentic AR primary target genes, identified three dominant AR‐binding motifs, two of them, termed motif 2 and 3, are enriched around the transcription start sites of nearby target genes. These two motifs also tend to cluster with each other. Motif 3 is highly similar to a known motif bound by AR (MA0007.1), whereas motif 2 is a potential novel AR‐binding motif. By analyzing the activity of this novel motif in an ARBS located in an intron of the *MLPH* gene, where it is 11 bases distant to a motif 3 element, we found that motif 2 alone could not mediate androgen regulation. It seems to be an auxiliary AR motif and play a modulatory role, fine‐tuning AR signaling.

Investigating an involvement of PCa risk SNPs in modulating AR signaling, we found that PCa GWAS SNPs and their proxies are highly over‐represented in the identified ARBSs. We have chosen rs11891426:T>G located in the novel auxiliary motif 2 in the *MLPH* AR enhancer to test the functional impact of this SNP in modulating AR transcriptional activity on the respective gene. Reporter gene assays showed a reduced AR transactivation activity when the major T allele was changed to the risk G allele, corroborating the role of this SNP and its host motif 2 in modulating AR regulation of MLPH expression. This role is similar to another SNP located in the TMPRSS2 enhancer that also reduces AR transactivation [Clinckemalie et al., 2013]. These findings indicate a potential function of PCa risk SNPs to promote tumorigenesis via modulating AR signaling. In agreement with the enrichment of PCa risk SNPs in ARBSs, recent efforts to prioritize genetic variants for downstream functional evaluation by overlapping risk SNPs with epigenetic marks and expression quantitative trait loci analysis identified genetic variants of which a majority is localized within promoter and enhancer regions [Han et al., 2015]. In a similar approach, Al Olama et al. (2015) defined a new index risk SNP in the *MLPH* gene, rs11891348:T>G, which is even closer to rs11891426:T>G in the ARBS than the old index SNP rs2292884:A>G.

Genotype–phenotype association analysis of rs11891426:T>G in benign and malignant prostate tissues revealed reduced expression of MLPH in the presence of a risk G allele. This is consistent with the reporter assay result, indicating that this SNP is negatively modulating androgen regulation of *MLPH* expression. Previous GWAS studies have identified two PCa susceptibility loci at 2q37.3 with the two SNPs, rs2292884:A>G and rs7584330:A>G, located either within or next to the *MLPH* gene [Haiman et al., 2011; Kote‐Jarai et al., 2011a, 2011b; Schumacher et al., 2011]. The risk G allele of rs2292884:A>G, which has a stronger linkage disequilibrium with rs11891426:T>G compared with rs7584330:A>G, also strongly correlated with reduced *MLPH* gene expression in human prostates. Along with the evidence of rs11891426:T>G as a putative risk SNP within the *MLPH*‐ARBS and the correlation of *MLPH* and *AR* expression in prostate tissue at protein and mRNA level, these data further imply a predisposition to PCa via modulating *MLPH* gene expression.

MLPH is a member of the exophilin subfamily of Rab effector proteins, an interaction partner of the small Ras‐related GTP‐ases Rab27A and Rab27B on one hand and of the motor protein myosin on the other [Fukuda et al., 2002]. The complex MLPH/Rab27/myosin is required for the polarized transport of melanosomes along the actin cytoskeleton in melanocytes [Matesic et al., 2001], for the pigmentation of the hair and skin [Menasche et al., 2000] and for the secretion pathway [Fukuda, 2013]. Little is known about a possible functional impact in carcinogenesis or tumor progression. Dysregulation of MLPH was found in several types of tumors, for example, lung cancer, meningiomas, and breast cancer [Fevre‐Montange et al., 2009; Pio et al., 2010; Thakkar et al., 2010; Molina‐Pinelo et al., 2014]. A very recent study found association of expression of *MLPH* (and some other genes) with nearby SNPs in prostate tissue [Penney et al., 2015]. In non‐small cell lung cancer MLPH mRNA was identified as a target of differentially expressed miRNAs [Molina‐Pinelo et al., 2014]. Interestingly, MLPH was found to be significantly overexpressed in estrogen receptor (ER) positive breast cancer suggesting a regulation of this protein by estrogen hormones [Thakkar et al., 2010].

In our study, we found both androgen regulation in PCa cells and significant positive correlations of MLPH and AR mRNAs and proteins in benign and malignant prostate tissue implying AR involvement in *MLPH* regulation *in vivo*. Most interestingly, correlation of MLPH expression to clinicopathological variables of patients revealed a clear correlation of higher expression with a favorable risk profile, including lower Gleason grades, lower pathological stages, smaller relative tumor mass, absence of biochemical tumor recurrence, and lower serum PSA. Solely, the correlation with lower patients’ age at diagnosis does not fit into this line. Taken together, the expression pattern of MLPH points toward a tumor‐suppressive function of MLPH, which is weakened in the presence of a rs11891426:T>G risk G allele by attenuation of androgen and AR‐regulated expression of MLPH.

Our work took advantage of next‐generation sequencing for genome wide profiling of ARBSs in the DUCaP PCa cell line that represents tumors harboring *ERG* gene rearrangements. We deciphered a possible regulatory mechanism of a PCa GWAS candidate gene, *MLPH*, and its link to the androgenome. The mechanism of *MLHP* regulation in benign and malignant prostate tissues identified a highly likely causative SNP, rs11891426:T>G, around 50 kb away from the originally associated PCa risk index SNPs. Higher expression of MLPH in the prostate tissue of cancer patients with a favorable risk profile provides evidence for a tumor‐suppressive function of this protein. Collectively, our study reveals that identifying the plausible causative SNP for a complex disease requires careful functional assessment of each SNP around the GWAS index SNPs. Further work is needed to clarify in‐depth its role in disease development and the function of MLPH in benign and malignant prostate cells.

*Disclosure statement*: The authors declare no conflict of interest.

## Supporting information

Supporting Information

Table s1
