# Supplementary material for: Putative Prostate Cancer Risk SNP in an Androgen Receptor‐Binding Site of the Melanophilin Gene Illustrates Enrichment of Risk SNPs in Androgen Receptor Target Sites
Source: Hum Mutat. 2015 Oct 19;37(1):52–64. doi: 10.1002/humu.22909 (PMC4715509; doi:10.1002/humu.22909)
Supplement: Supplementary file 1 — Supporting Information [file HUMU-37-52-s001.pdf]

Supporting Information for the article

Putative Prostate Cancer Risk SNP in an AR Binding Site of the Melanophilin Gene Illustrates Enrichment of Risk SNPs in AR Target Sites

Huajie Bu[1,2*], Narisu Narisu[3*], Bettina Schlick[1,4] , Johannes Rainer[5,11], Thomas Manke[6,9], Georg Schäfer[1,7], Lorenza Pasqualini[1], Peter Chines[3], Michal R. Schweiger[6,10], Christian Fuchsberger[8,11], Helmut Klocker[1]

Supp. Table S1 is available as a separate Excel file under the Supporting Information for this article.

Supp. Table S2. List of prostate cancer risk SNPs and their proxy risk SNPs overlapping with AR binding sites

Nr.	SNP	Chr	Position	GWAS index SNP	r2 with GWAS index SNP	Reported gene(s)	Ref.
1	rs56005245	chr8	128182608	rs1016343	0.90		
2	rs1048739	chr2	85618479	rs10187424	0.53	GGCX, VAMP8, VAMP5, RNF181	[1]
3	rs1048740	chr2	85618477	rs10187424	0.55	GGCX, VAMP8, VAMP5, RNF181	[1]
4	rs1446668	chr2	85618471	rs10187424	0.85	GGCX, VAMP8, VAMP5, RNF181	[1]
5	rs2289972	chr2	85620056	rs10187424	0.57	GGCX, VAMP8, VAMP5, RNF181	[1]
6	rs79509278	chr2	85618952	rs10187424	0.57	GGCX, VAMP8, VAMP5, RNF181	[1]
7	rs7929962	chr11	68742159	rs10896449	1.00	Intergenic	[4]
8	rs1735545	chr3	129563950	rs10934853	0.74	Intergenic	[2]
9	rs2659685	chr3	129605086	rs10934853	0.64	Intergenic	[2]
10	rs6777054	chr3	129428039	rs10934853	0.91	Intergenic	[2]
11	rs9814834	chr3	129420827	rs10934853	0.91	Intergenic	[2]
12	rs9847576	chr3	129420335	rs10934853	0.91	Intergenic	[2]
13	rs12649976	chr4	95763308	rs12500426	0.72	PDLIM5	[3]
14	rs13110828	chr4	95793234	rs12500426	0.64	PDLIM6	[3]
15	rs6853490	chr4	95763741	rs12500426	0.79	PDLIM7	[3]
16	rs13256300	chr8	23558533	rs1512268	0.92	NR	[5]
17	rs13256366	chr8	23558605	rs1512268	0.92	NR	[5]
18	rs71504412	chr8	23558910	rs1512268	0.87	NR	[5]
19	rs74305597	chr8	128182646	rs16901979	1.00	Intergenic	[2]
20	rs7824451	chr8	128183643	rs16901979	1.00	Intergenic	[2]
21	rs72712310	chr8	128402747	rs16902094	0.98	Intergenic	[2]
22	rs72712344	chr8	128411808	rs16902094	0.92	Intergenic	[2]
23	rs7689746	chr4	95793146	rs17021918	0.53	PDLIM5	[3]
24	rs1859962	chr17	66620348	rs1859962	1.00	Intergenic	[6]
25	rs8071558	chr17	66619268	rs1859962	1.00	Intergenic	[6]
26	rs8072254	chr17	66619411	rs1859962	1.00	Intergenic	[6]
27	rs11883500	chr2	238099173	rs2292884	0.61	MLPH	[6]
28	rs11890255	chr2	238099344	rs2292884	0.61	MLPH	[6]
29	rs11890307	chr2	238099444	rs2292884	0.61	MLPH	[6]
30	rs11891426	chr2	238099441	rs2292884	0.53	MLPH	[6]
31	rs7558685	chr2	238099682	rs2292884	0.51	MLPH	[6]
32	rs7598035	chr2	238099681	rs2292884	0.51	MLPH	[6]
33	rs2611471	chr10	51173565	rs3123078	0.82	NR	[3]
34	rs7090326	chr10	51173381	rs3123078	0.82	NR	[3]
35	rs339331	chr6	117316745	rs339331	1.00	GPRC6A,RFX6	[7]
36	rs4946205	chr6	117352082	rs339331	0.52	GPRC6A,RFX6	[7]
37	rs63749614	chr6	117363699	rs339331	0.91	GPRC6A,RFX6	[7]
38	rs657963	chr6	117357944	rs339331	0.52	GPRC6A,RFX6	[7]
39	rs674621	chr6	117363711	rs339331	0.91	GPRC6A,RFX6	[7]
40	rs675495	chr6	117363892	rs339331	0.91	GPRC6A,RFX6	[7]
41	rs6901971	chr6	117221096	rs339331	0.78	GPRC6A,RFX6	[7]
42	rs7770158	chr6	117351067	rs339331	0.52	GPRC6A,RFX6	[7]

Nr.	SNP	Chr	Position	GWAS index SNP	r2 with GWAS index SNP	Reported gene(s)	Ref.
43	rs7774506	chr6	117351369	rs339331	0.52	GPRC6A,RFX6	[7]
44	rs10099413	chr8	128591245	rs4242384	0.75	Intergenic	[6]
45	rs11986220	chr8	128600871	rs4242384	1.00	Intergenic	[6]
46	rs11988857	chr8	128601055	rs4242384	0.84	Intergenic	[6]
47	rs7812429	chr8	128589355	rs4242384	1.00	Intergenic	[6]
48	rs7814837	chr8	128591384	rs4242384	1.00	Intergenic	[6]
49	rs17464492	chr8	128412048	rs445114	0.65	Intergenic	[6]
50	rs4907792	chrX	51265523	rs5945619	0.99	Intergenic	[6]
51	rs11043132	chr11	2189200	rs7127900	1.00	IGF2, IGF2AS, INS, TH	[3]
52	rs11043133	chr11	2189241	rs7127900	1.00	IGF2, IGF2AS, INS, TH	[3]
53	rs11043135	chr11	2189272	rs7127900	1.00	IGF2, IGF2AS, INS, TH	[3]
54	rs6578999	chr11	2188350	rs7127900	0.99	IGF2, IGF2AS, INS, TH	[3]
55	rs6579000	chr11	2188618	rs7127900	1.00	IGF2, IGF2AS, INS, TH	[3]
56	rs6579001	chr11	2188632	rs7127900	1.00	IGF2, IGF2AS, INS, TH	[3]
57	rs6579002	chr11	2188805	rs7127900	1.00	IGF2, IGF2AS, INS, TH	[3]
58	rs6579003	chr11	2188886	rs7127900	1.00	IGF2, IGF2AS, INS, TH	[3]
59	rs7107385	chr11	2171380	rs7127900	0.61	IGF2, IGF2AS, INS, TH	[3]
60	rs7123180	chr11	2188848	rs7127900	1.00	IGF2, IGF2AS, INS, TH	[3]
61	rs7123299	chr11	2188920	rs7127900	1.00	IGF2, IGF2AS, INS, TH	[3]
62	rs7123312	chr11	2188935	rs7127900	1.00	IGF2, IGF2AS, INS, TH	[3]
63	rs7123418	chr11	2188954	rs7127900	1.00	IGF2, IGF2AS, INS, TH	[3]
64	rs7125284	chr11	2171216	rs7127900	0.61	IGF2, IGF2AS, INS, TH	[3]
65	rs12793759	chr11	68731131	rs7130881	0.91	Intergenic	[6]
66	rs60676526	chr17	44809379	rs7210100	1.00	ZNF652	[8]
67	rs73338357	chr17	44624978	rs7210100	1.00	ZNF652	[8]
68	rs12474519	chr2	238041751	rs7584330	0.84	MLPH	[1]
69	rs13386290	chr2	238057875	rs7584330	0.88	MLPH	[1]
70	rs6737914	chr2	238058173	rs7584330	0.96	MLPH	[1]
71	rs6740722	chr2	238058027	rs7584330	0.50	MLPH	[1]
72	rs80151891	chr2	238063565	rs7584330	0.50	MLPH	[1]
73	rs112162741	chr12	51560766	rs902774	1.00	KRT8, EIF4B, TENC1	[6]
74	rs4919737	chr12	51557062	rs902774	0.84	KRT8, EIF4B, TENC2	[6]
75	rs4919740	chr12	51557238	rs902774	0.83	KRT8, EIF4B, TENC3	[6]
76	rs4919743	chr12	51595851	rs902774	0.82	KRT8, EIF4B, TENC4	[6]
77	rs73108429	chr12	51557938	rs902774	0.84	KRT8, EIF4B, TENC5	[6]
78	rs902772	chr12	51560040	rs902774	1.00	KRT8, EIF4B, TENC6	[6]
79	rs902773	chr12	51560046	rs902774	1.00	KRT8, EIF4B, TENC7	[6]
80	rs902774	chr12	51560171	rs902774	1.00	KRT8, EIF4B, TENC8	[6]

References:

[1] Kote-Jarai, Z., et al. Nat Genet, 2011. **43**(8): p. 785-91.

[2] Gudmundsson, J., et al. Nat Genet, 2009. **41**(10): p. 1122-6.

[3] Eeles, R.A., et al. Nat Genet, 2009. **41**(10): p. 1116-21.

[4] Thomas, G., et al. Nat Genet, 2008. **40**(3): p. 310-5.

[5] Cheng, Y., et al. Prostate, 2009. **69**(3): p. 327-35.

[6] Schumacher, F.R. Hum Mol Genet, 2011. **20**(19): p. 3867-75.

[7] Takata, R., et al. Nat Genet, 2010. **42**(9): p. 751-4.

[8] Haiman, C.A., et al. Nat Genet, 2011. **43**(6): p. 570-3.

Supp. Table S3. Association of MLPH genotypes with clinicopathological characteristics

Variable		rs11891426 (no.) TT	TG & GG	P-value	rs2292884 (no.) AA	AG & GG	P value	rs7584330 (no.) AA	AG & GG	P value
Age	≤ 60 (42)	29	13		24	18		23	19	
	> 60 (65)	51	14	0.27	42	23	0.44	45	20	0.13
Total PSA (g)	≤ 4 (31)	22	9		22	9		19	12	
	> 4 (74)	55	19	0.72	43	31	0.22	48	26	0.73
free PSA (%)	≤ 13.05 (42)	30	12		22	20		26	16	
	> 13.05 (41)	32	9	0.49	28	13	0.14	25	16	0.93
Tumor volume (ml)	≤ 1.08 (12)	9	3		9	3		8	4	
	> 1.08 (20)	18	2	0.26	15	5	1.00	15	5	0.61
Gleason score	≤ 3+4 (57)	42	15		32	25		32	25	
	≥ 4+3 (51)	38	13	0.92	34	17	0.26	36	15	0.12
Pathological stage	T2a+T2c (60)	45	15		33	27		32	28	
	T3a+T2b+T4 (48)	35	13	0.81	33	15	0.15	36	12	**0.021**
PSA recurrence	Yes (30)	20	10		15	15		19	11	
	No (42)	34	8	0.17	28	14	0.16	26	16	0.90
Relative estimated tumor volume (%)	≤ 10% (39)	30	9		25	14		22	17	
	> 10% (33)	24	9	0.68	21	12	0.97	25	8	0.086

Significant P-values are highlighted in bold; borderline significant P-values are underlined

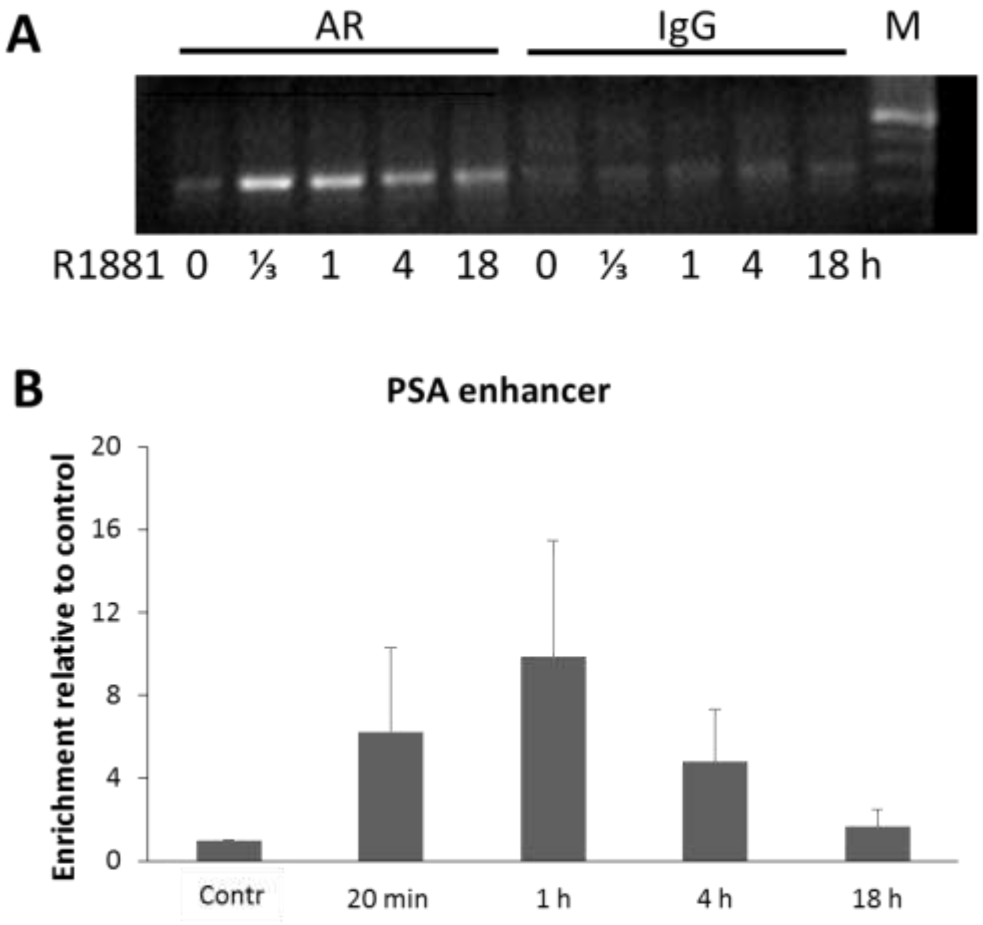

Supp. Figure S1. Dynamics of AR binding to the PSA enhancer. DUCaP cells were stimulated with 1 nM R1881 for the indicated time points, harvested and subuected to ChIP isolation of AR bound DNA. Rabbit IgG was used as antibody control. **A:** PCR with AR-precipitated samples were used to visualize the dynamics of AR recruitment to its target gene PSA. M: DNA ladder. **B:** QPCR was used to quantify the androgen stimulated AR enrichment to the binding site relative to control vehicle-treated samples (Contr). Values in (**B**) represent the mean values and standard deviations of at least three independent experiments.

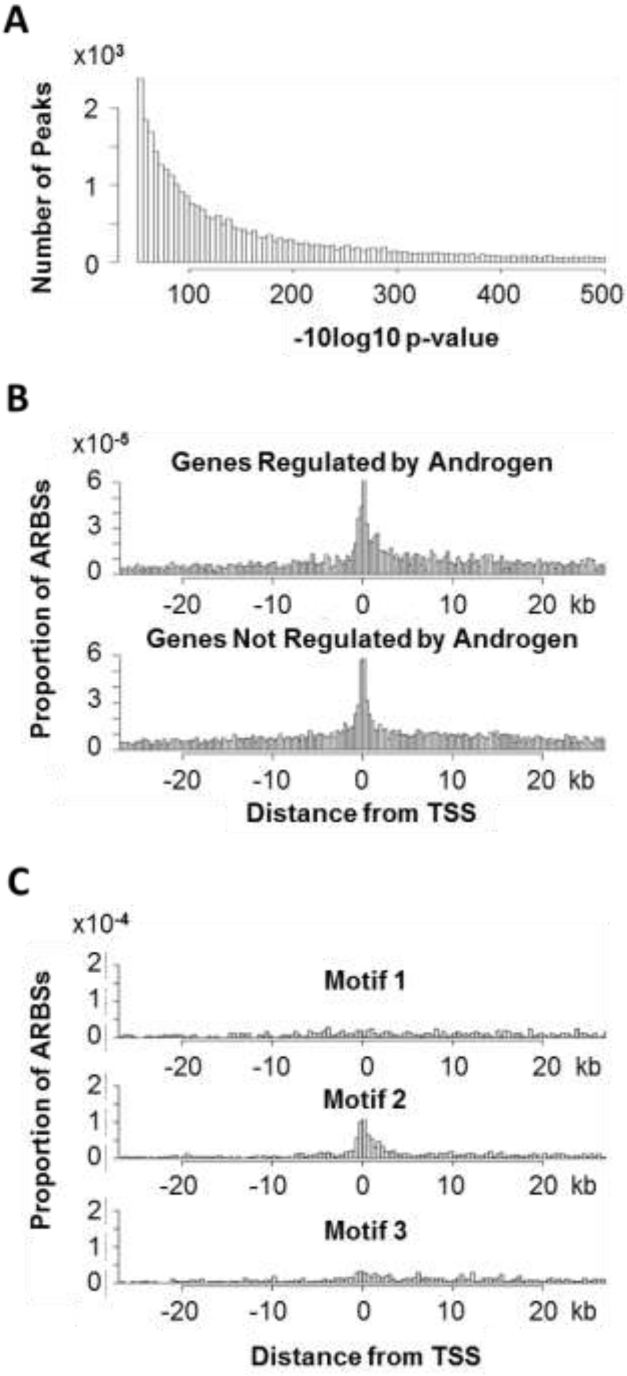

Supp. Figure S2. Characterization of AR binding sites. **A:** Illustration of MACS calculated P values of the AR binding peaks. **B:** Illustration of the distance of ARBSs to the transcription start sites (TSSs) of their adjacent genes. The upper and lower panels represent the distance of ARBSs to the TSS of the primary AR target genes as defined in legend to Figure 1 (upper panel) and to the TSS of all other genes (lower panel), respectively. **C:** Illustration of the distance of the identified 3 putative ARE motifs to the TSS of the adjacent genes.

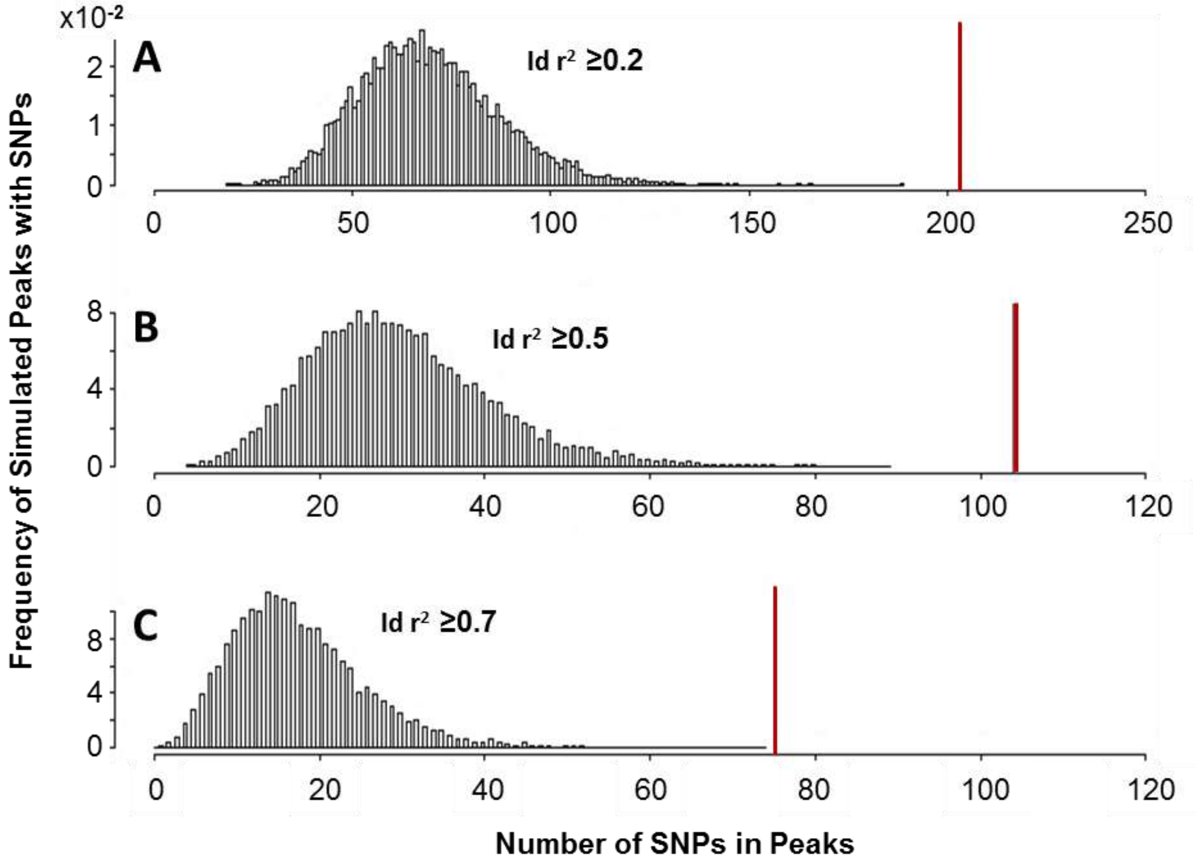

Supp. Figure S3. Simulation analysis of ChIP-seq peaks. Number of reported GWAS PCa risk SNPs and their proxies with simulated AR binding sites. **A:** All proxy SNPs with linkage disequilibrium (ld) of $r^2 \geq 0.2$ with the reported SNPs were included and the simulation of ARBSs was repeated 10,000 times to determine random co-localization. **B:** All proxies with ld of $r^2 \geq 0.5$ were included. **C:** All proxies with ld $r^2 \geq 0.7$ were included. The vertical red line indicates the actual number of SNPs in the identified ARBSs and demonstrates the enrichment of risk SNPs in AR binding sites.

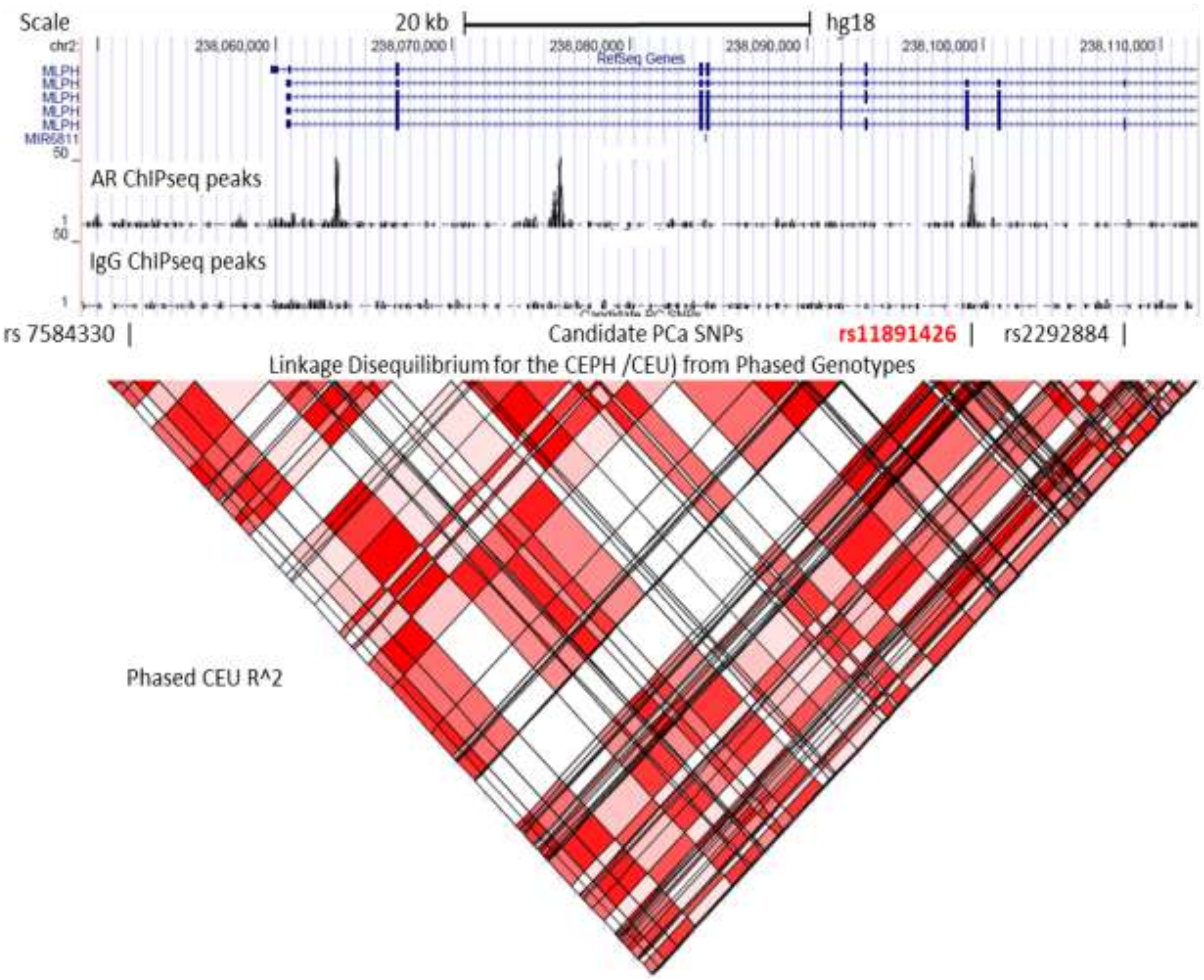

Supp. Figure S4. Id structure of SNPs in the melanophilin gene. The PCa risk index SNP rs7584330 and the potential PCa risk SNPs rs11891429 and rs2294884 are indicated in relation to the melanophilin gene and the AR ChiP-seq peaks. From top to bottom: refseq gene, ARBS peaks, IgG peaks (control), potential PCa risk SNPs and Id structure.

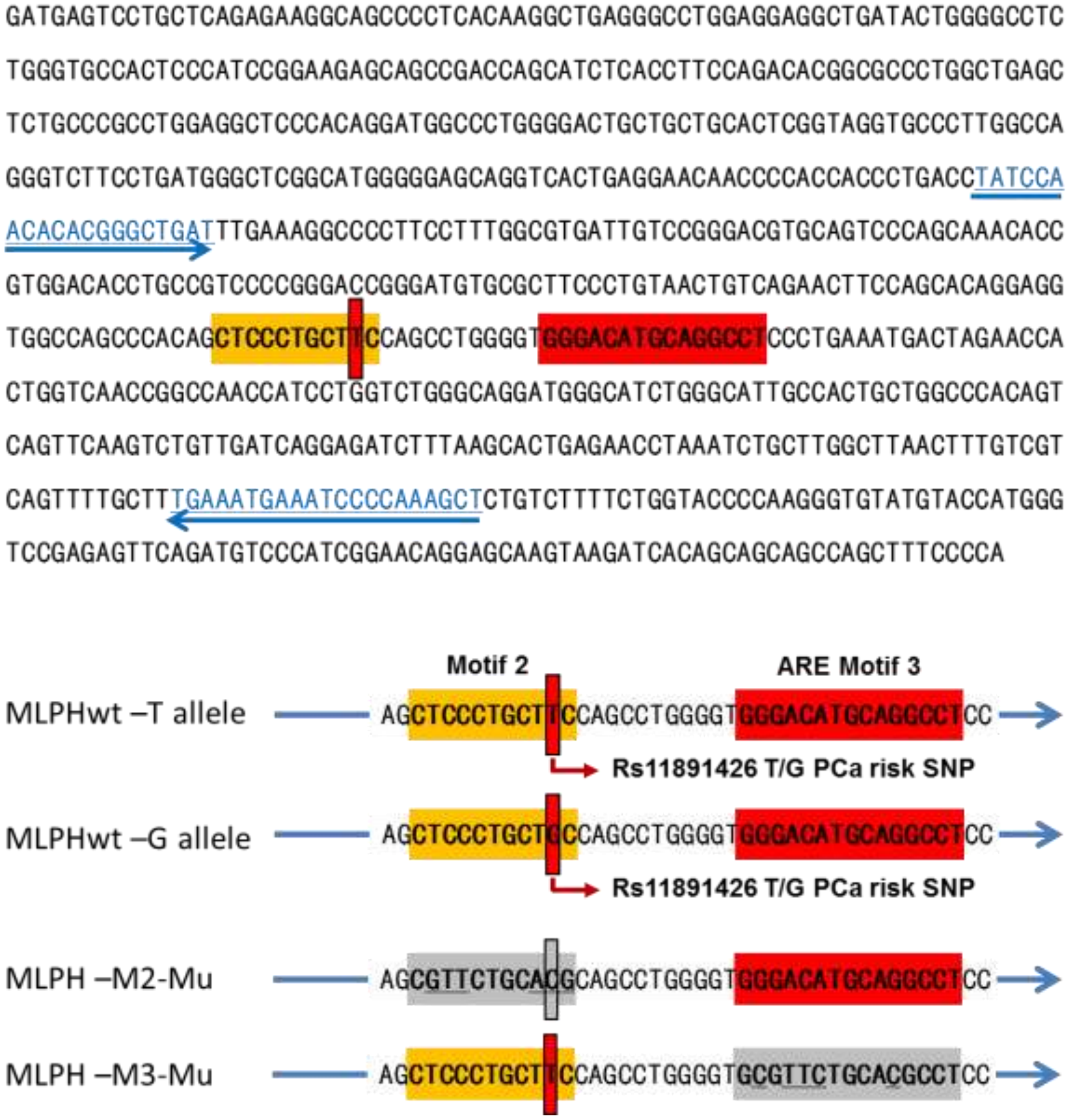

Supp. Figure S5. AR binding site in the 7th intron of the MLPH gene. **A:** The DNA sequence of the MLPH gene ARBS is shown. The putative ARE motifs 2 and 3, the rs11891426 SNP within the motif 2 ARE element, as well as the primers used for cloning and sequencing of the ARBS are indicated. **B:** Illustration of MLPH ARBS reporter gene constructs used. The wild-type construct containing the SNP rs11891426 major T allele was designated as MLPH-wt, the constructs with the SNP site Rs11891426 mutated to the minor G allele as MLPH-G. The mutated motif 3 or motif 2 ARE reporter vectors were designated as MLPH-M3-mut or MLPH-M2-mut, respectively.

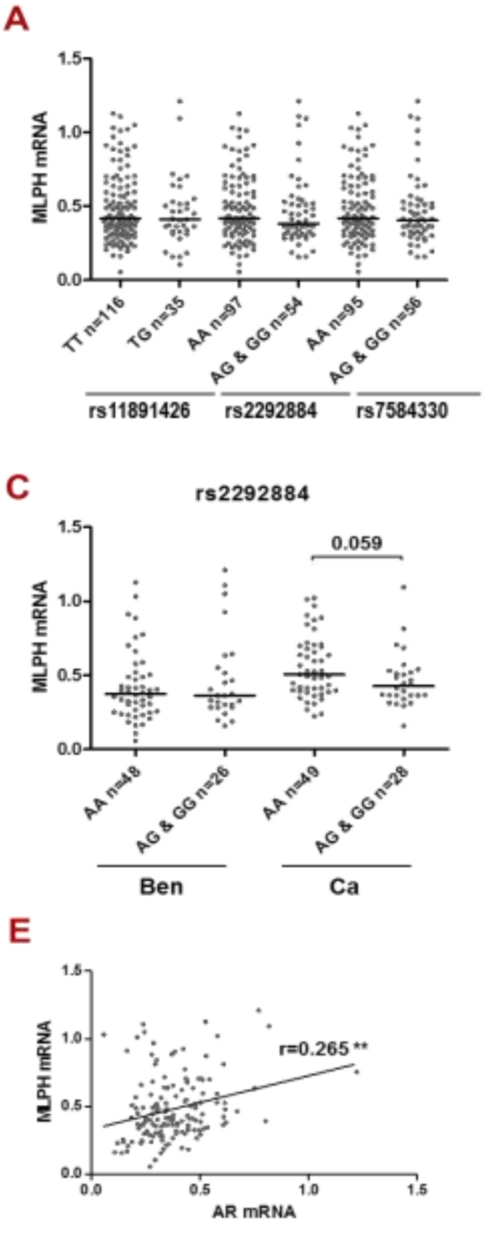

Supp. Figure S6. Correlation of MLPH mRNA expression to the SNP genotypes and to AR expression in PCa tissue specimens. Expression of MLPH and AR transcripts was analysed by real-time RT-PCR in RNA samples extracted from benign and malignant areas of frozen radical prostatectomy specimens (n=77). The relative transcript expression levels were calculated in relation to the house keeping gene TBP and further calibrated to an inter-assay and inter-plate control sample (cDNA mix of 3 benign and 3 malignant tissue sections). **A**-**D**: Scatter plot of MLPH mRNA expression stratified according to the genotypes of rs11891426, rs2292884 and rs7584330 (**A**), tissue types and genotype category of rs11891426 (**B**), rs2292884 (**C**) and rs7584330 (**D**). **E:** Benign (Ben) and cancer (Ca) tissue types. **F:** Scatter plot of MLPH versus AR mRNA demonstrating the correlated expression of AR and MLPH mRNAs in prostate tissue samples. Welch's corrected unpaired t-test was used for the analysis of differences between the groups. * P < 0.05, ** P < 0.01 and *** P < 0.001.

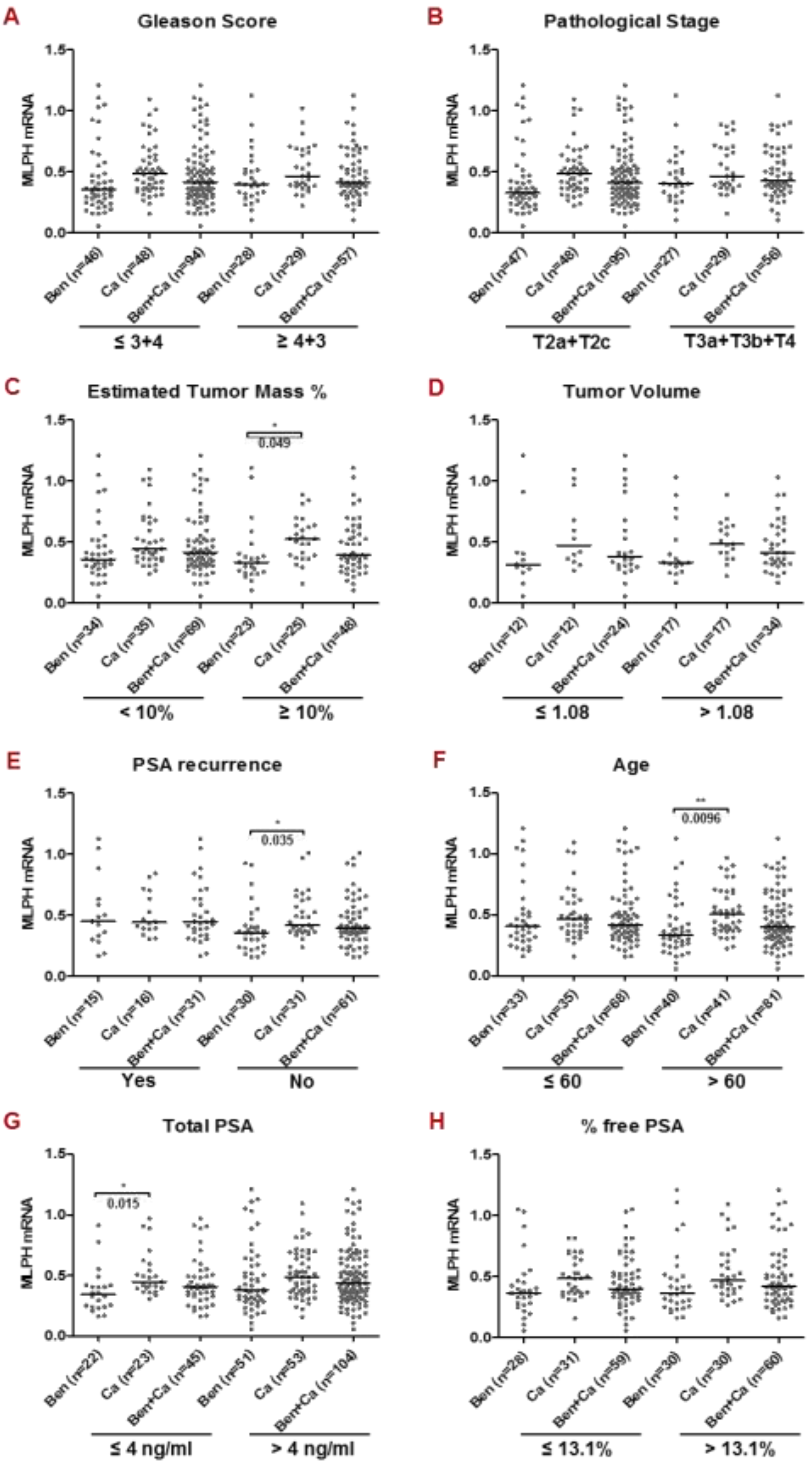

Supp. Figure S7. Correlation of *MLPH* mRNA expression to clinicopathological tumor characteristics. MLPH protein ex-pression was deter-mined by qPCR and samples were stratified according to A: Gleason score, B: Histopatho-logical stage, C: Percent estimated tumor mass, D: Tumor volume, E: PSA recurrence (bio-chemical recurrence), F: Age at tumor diagnosis, G: Total PSA serum level and H: Percent free PSA. The Welch's corrected unpaired t-test was used for the analysis of differences between the groups. * $P < 0.05$, ** $P < 0.01$ and *** $P < 0.001$.
